# Transcriptomic dissection reveals wide spread differential expression in chickpea during early time points of *Fusarium oxysporum* f. sp. *ciceri* Race 1 attack

**DOI:** 10.1371/journal.pone.0178164

**Published:** 2017-05-25

**Authors:** Sumanti Gupta, Anirban Bhar, Moniya Chatterjee, Amartya Ghosh, Sampa Das

**Affiliations:** Division of Plant Biology, Bose Institute, Centenary Campus, P 1/12, CIT Scheme, VII-M, Kankurgachi, Kolkata, West Bengal, India; Tallinn University of Technology, ESTONIA

## Abstract

Plants’ reaction to underground microorganisms is complex as sessile nature of plants compels them to prioritize their responses to diverse microorganisms both pathogenic and symbiotic. Roots of important crops are directly exposed to diverse microorganisms, but investigations involving root pathogens are significantly less. Thus, more studies involving root pathogens and their target crops are necessitated to enrich the understanding of underground interactions. Present study reported the molecular complexities in chickpea during *Fusarium oxysporum* f. sp. *ciceri* Race 1 (Foc1) infection. Transcriptomic dissections using RNA-seq showed significantly differential expression of molecular transcripts between infected and control plants of both susceptible and resistant genotypes. Radar plot analyses showed maximum expressional undulations after infection in both susceptible and resistant plants. Gene ontology and functional clustering showed large number of transcripts controlling basic metabolism of plants. Network analyses demonstrated defense components like peptidyl cis/trans isomerase, MAP kinase, beta 1,3 glucanase, serine threonine kinase, patatin like protein, lactolylglutathione lyase, coproporphyrinogen III oxidase, sulfotransferases; reactive oxygen species regulating components like respiratory burst oxidase, superoxide dismutases, cytochrome b5 reductase, glutathione reductase, thioredoxin reductase, ATPase; metabolism regulating components, myo inositol phosphate, carboxylate synthase; transport related gamma tonoplast intrinsic protein, and structural component, ubiquitins to serve as important nodals of defense signaling network. These nodal molecules probably served as hub controllers of defense signaling. Functional characterization of these hub molecules would not only help in developing better understanding of chickpea-Foc1 interaction but also place them as promising candidates for resistance management programs against vascular wilt of legumes.

## Introduction

Legumes are well known for their nutritive value consisting of easily digestible proteins [1].Besides, their ability to form nitrogen fixing nodules with Gram negative rhizobia further adds to their importance. But knowledge on how these plants encounter with harmful pathogens is still limited. Substantial researches have been carried out on interactions involving model legume plants like *Medicago* and *Lotus* and soil inhabiting pathogens [2,3], but reports on crop plants that are also exposed to dreadful attacks by diverse members of soil pathogens are significantly inadequate. Advancement of biotechnological tools and their applications have added remarkably to the genome sequencing and annotation projects with draft genome sequences being available for many important crop legumes like soybean, pigeonpea, chickpea etc. [4,5,6,7]. However, with the exception of soybean, researches on other crop legumes are gradually increasing [8].

Chickpea tops the Indian list of important pulse legumes [9]. But, vascular wilt disease of chickpea is known to account for 10–15% annual yield loss, which escalates to total loss under specific edaphic and environmental conditions well suited for the replication and establishment of Foc. Amongst 8 pathovars (0, 1B/C. 1–6), Race1 has received prime scientific attention due to its widespread distribution, thus causing maximum damage [10]. *Fusarium* wilt was known to be primarily managed by conventional breeding programs. But pathogenic variability and mutability have led to the breakdown of natural resistance over prolonged periods [11]. Besides, long term application of chemical fungicides has also raised serious social concern regarding health and environmental safety [12]. Hence, a safe and sustainable alternative is still on the lookout for managing *Fusarium* wilt of chickpea.

Previous reports on chickpea-Foc1 interaction documented transcriptomic alterations during pathogen attack [13–16]. Besides, biochemical investigations reported induction of several stress induced marker isozymes [17]. Genetic mapping and linkage analyses identified chromosome loci linked to *Fusarium* resistance in chickpea as well as other legumes [18–19]. Even then, the knowledge of sequential events and involvement of resistant gene (s) in mediating the signaling cascade is still obscure. Studies conducted by the present group made attempts to delineate the host responses upon pathogen assault in steps and phase wise manner. Initial studies identified the temporal sequences of pathogen progression and their external manifestations [14]. Following studies figured out the primary metabolism to be the initial target of the wound inducing Foc1 that was found to overpower the susceptible host [15]. Reactive oxygen species were identified as to be the initial triggering factor igniting the entire defense signaling cascade which was found to be well coordinated with internal cellular transporters and transcription factors [16]. In a parallel attempt proteomic analyses were conducted to identify the differential defense responsive proteins mediating the entire signaling sequences at early time points of pathogen invasion [20]. With all the results taken together the chickpea-Foc1 case study has undoubtedly brought forth several significant results relating to the understanding of the complex disorder of vascular wilt but, many hubs of *in planta* signaling network yet remains incomprehensible that necessitates more transcriptomic analytical results. Although, large body of information indicates towards simultaneous participation of many intricate metabolic events but lack of fully annotated host genome sequence fails to plug the gap in this particular defense network. Moreover, wide genomic diversification across model legumes and crop legumes fails to completely transfer the knowledge of metabolic events from model plants to crop plants [21]. Thus, interactional case studies of crop legumes and pathogens demand individualistic approach.

The advent of next generation sequencing tools and techniques has made a paradigm shift in the field of functional genomics as it generates large data sets. In present study RNA-seq was performed at early time point (48 hours as pointed out to be crucial for the case study) upon Foc1 invasion and differential transcriptomic dissection was performed. The analyses revealed induction as well as suppression of several defense responsive transcripts. Finally attempts were made to map the defense responsive components in an inter-connected defense regulatory network and identify the regulatory hubs that presumably control the entire defense signaling cascade in chickpea during Foc 1 attack.

## Results

### Analyses of sequence quality, read assembly and transcript annotation

**Fig 1:**
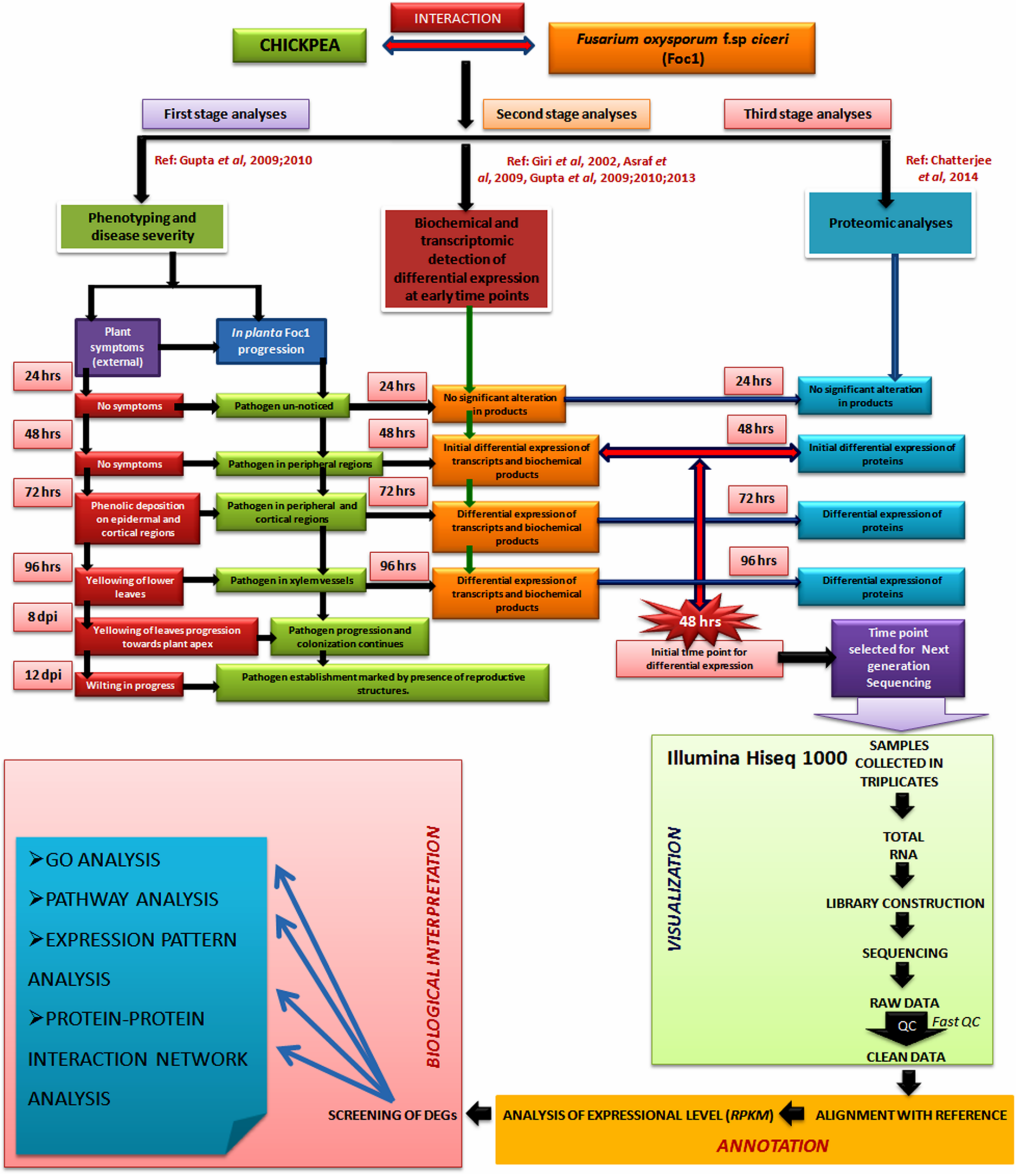
Schematic representation describing the rationale behind the work plan and NGS analysis work flow for the whole transcriptomic. Upper panel of the Flow diagram depicts 48h as crucial time point for differential expression of transcripts in chickpea after Foc1 infection and lower panel describes the NGS work flow and its downstream analyses.

**Fig 2:**
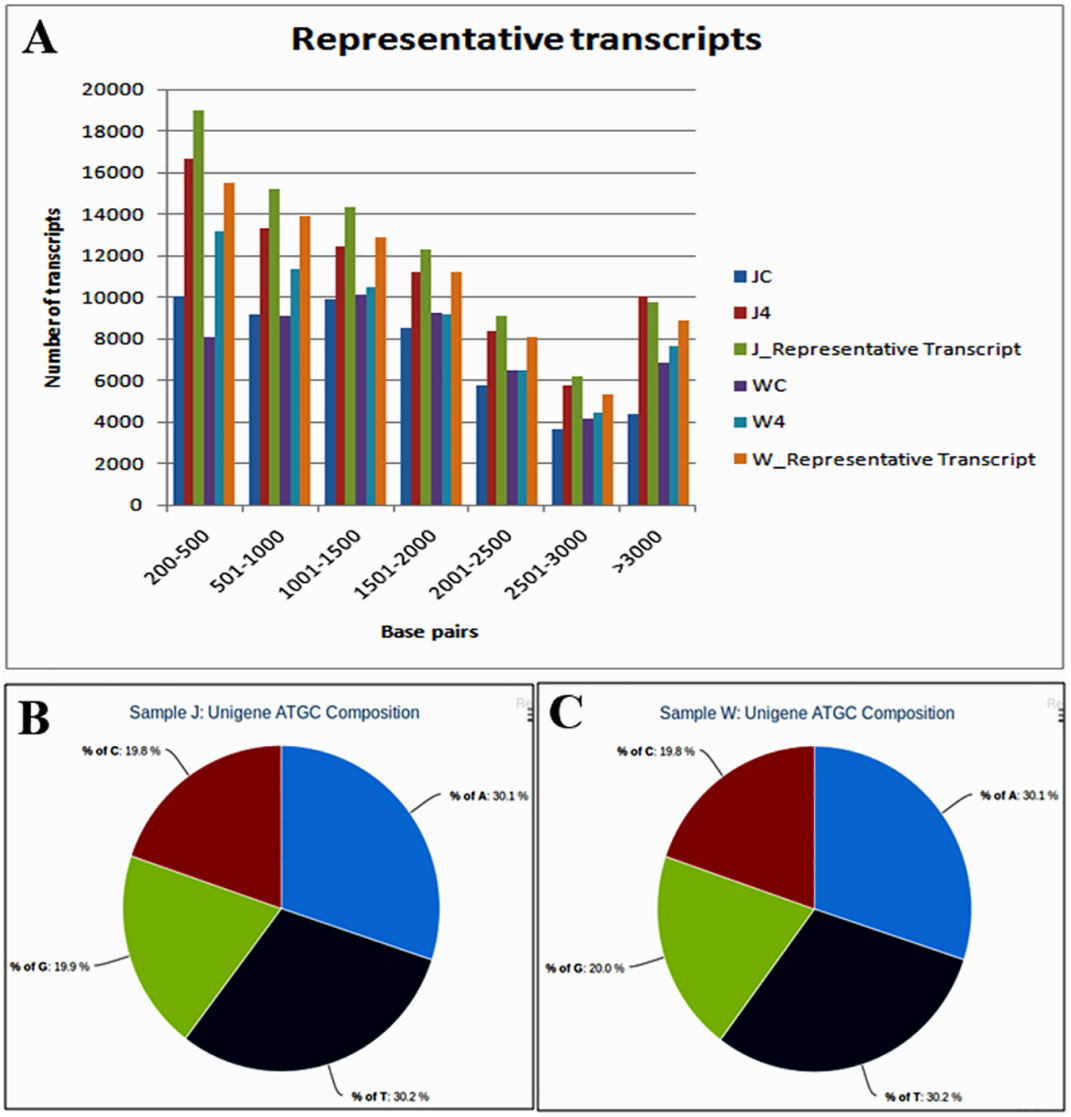
Graphical presentation of representative transcripts of susceptible (JG62) and resistant (WR315) chickpea and their ATGC distribution. **A**. Graph represents the base pair distribution of transcripts of JG62 (J4 and JC) and WR315 (W 4 and WC) with their representative transcripts. Pie chart represents unigene ATGC cluster of samples **B**. susceptible (JG62) and **C**. resistant (WR315) chickpea genotypes respectively.

Our previous studies zeroed on the time point of 48h that showed significant transcriptomic and proteomic alterations [15,16,20] (Fig 1, S1 Fig). Next generation sequencing analyses was performed on sample collected at 48h post inoculation with Foc1. Paired end sequence data was deposited to National Centre for Biotechnology Information’s (NCBI) Short Read Archive database under the accession number of **SRP041784** and BioProject ID **PRJNA246444**. Following adapter trimming 132.55 million, 89.6 million, 77.84 million, 89.86 million filtered reads were obtained for infected JG62 (represented by J4), uninfected JG62 (represented as JC), infected WR315 (represented as W4), uninfected WR315 (represented as WC), respectively. High quality (>Q20) bases were more than 96% for all the samples with low non ATGC characters (0.09%) for all the samples (S1 Table, S2 Fig). Filtered reads when assembled into contigs generated 79375 for J4, 45341 for JC, 59828 for W4 and 58650 for WC number of contigs (S2 Table, S3 Fig). Contigs were further assembled into transcripts generating 77770 transcripts for J4, 51366 transcripts for JC, 62713 transcripts for W4 and 53993 transcripts for WC respectively (S3 Table, S4 Fig). Representative transcripts (RT) after clustering contained 85915 transcripts for J and 75626 transcripts for W (S4 Table). Length and distribution of transcripts along with representative transcript are provided in Fig 2A. RTs of both J and W were found to be AT rich (60.33% for J and 60.20% for W) (Fig 2B). Amongst the transcripts generated, annotations were provided to 35597 for J4, 31726 for JC, 31636 for W4 and 35190 for WC, respectively. 50% identity and 40% query coverage was used as cutoff for annotating the transcripts (S5 Table).

### Differentially expressed transcript

**Fig 3:**
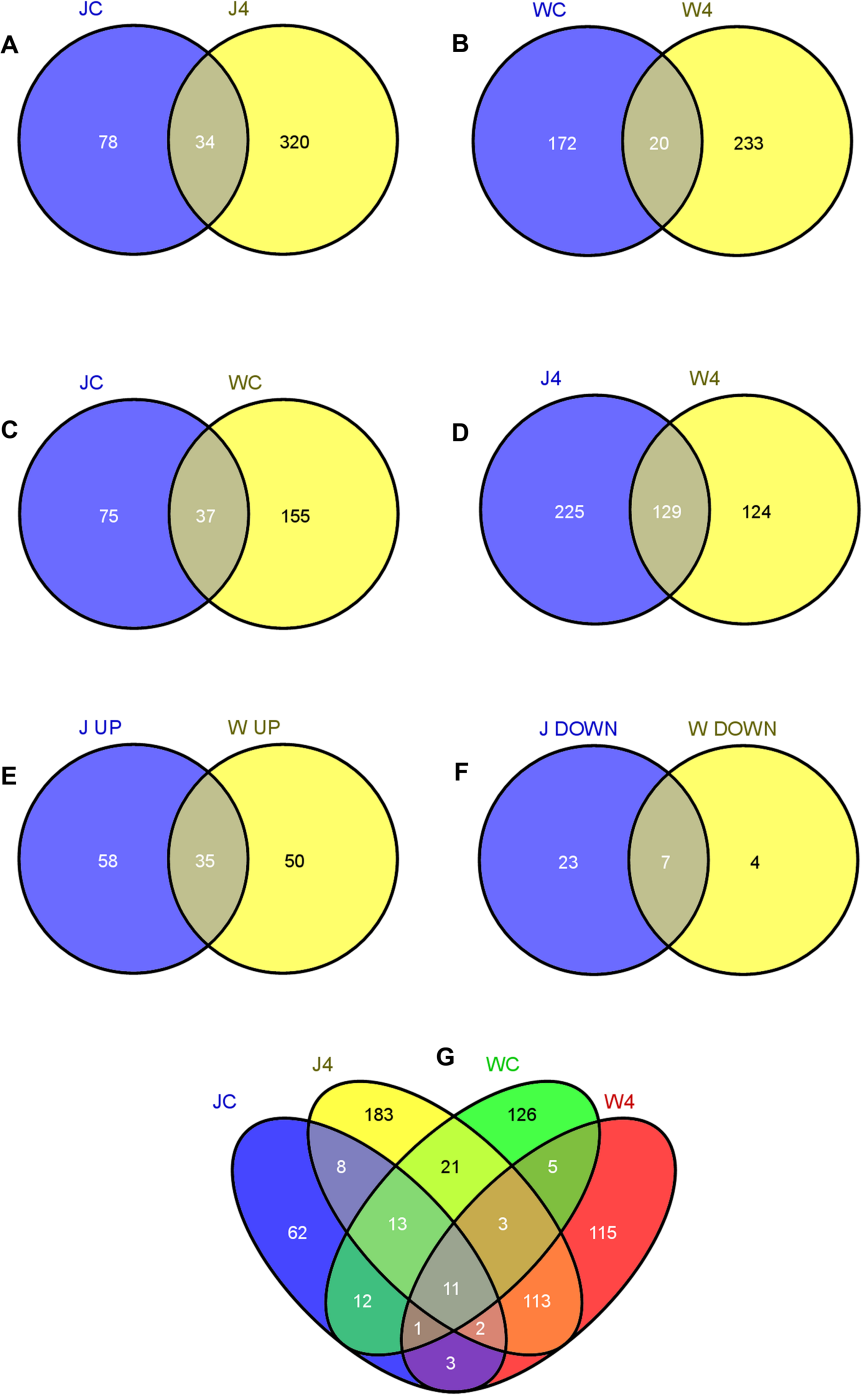
Venn diagram showing inter-distribution of transcripts. Distribution of transcripts between **A.** JC (uninduced susceptible) and J4 (induced susceptible) **B.** WC (uninduced resistant) and W4 (induced resistant) **C**. JC and WC, **D**. J4 and W4, **E**. Distribution of upregulated transcripts between J UP (upregulated in induced susceptible when compared to uninduced susceptible i.e comparing J4 and JC) and W UP (upregulated in induced resistant when compared to uninduced resistant i.e comparing W4 and WC) **F.** Distribution of down regulated transcripts between J Down (down regulated in induced susceptible when compared to uninduced susceptible i.e comparing J4 and JC) and W Down (down regulated in induced resistant when compared to uninduced resistant i.e. comparing W4 and WC). **G.** Distribution of transcripts between JC, J4, WC, W4.

Number of significant differential transcripts (with Q values) between J4 and JC were 2090 while between W4 and WC were 881 (S6 Table and S7 Table). Fig 3 demonstrates the occurrence of differential transcripts between samples. Out of total 466 differentially characterized transcripts, 320 were obtained from J4, 78 of JC, while 34 transcripts were common for both (Fig 3A). In case of W4 when compared to WC, 233 were generated from W4, 172 for WC, while 20 transcripts were common for both (Fig 3B). When comparison was made between JC and WC, 155 was found from WC, 75 from JC and 37 were in common (Fig 3C). Similarly, while comparing J4 and W4, 124 were obtained from W4, 225 from J4 and 129 were common for both W4 (Fig 3D). While comparing total up regulated transcripts, 58 were upregulated between JC and J4; and 50 were upregulated between WC and W4. 35 were commonly upregulated for both sample sets (JC vs J4 and WC vs W4) (Fig 3E, S8 Table, S5 Fig). As for down regulated only 4 were obtained between WC and W4, 23 between JC and J4, and 7 were found to be commonly downregulated for both sample sets (JC vs J4 and WC vs W4) (Fig 3F, S8 Table, S5 Fig). Comparison between all the four samples J4, JC, W4 and WC showed 183 transcripts appeared solely from J4, 62 from JC, 115 from W4 and 156 from WC. 21 transcripts were common for J4 and WC, 8 were common for JC and J4, and 13 were common for JC, J4, and WC. 5 transcripts were common for WC and W4, 3 were common for WC, W4, and JC, 11 were common for all the four samples. 12 transcripts were common for JC and WC, 1 for JC, WC and W4, 3 were common for JC and W4, and 2 were common for J4, JC and W4, while 113 were common for J4 and W4, respectively.

**Fig 4:**
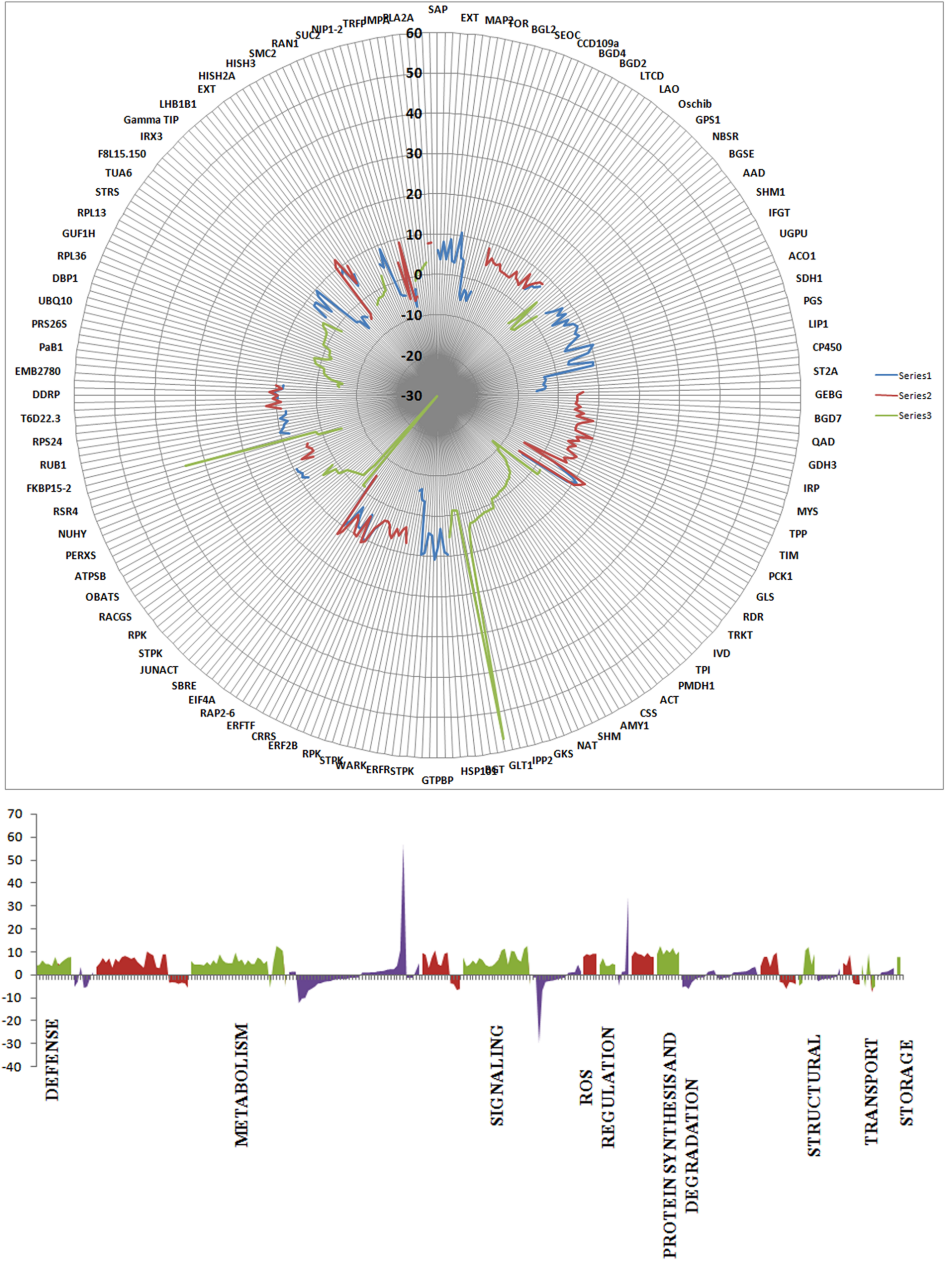
Radar plot representing the distribution of differential expression of transcripts. Upper panel represents total distribution of transcripts between JC, J4, WC, and W4. Lower panel represents distribution of transcripts according to their biological functions.The blue line explains the fold change value of transcripts that are found only in susceptible JG62 plants whereas red line demonstrates the expression values of transcripts found exclusively in resistant WR315 plants. The region where red and blue lines overlap with one another explains transcripts that are found in both JG62 and WR315 plants. Green line independently explains the transcripts that are found to be induced in JG62 and WR315 plants only after infection.

Radar plot analysis explains the distribution of fold change of the entire transcripts as obtained from the base mean values of each transcript. The blue line explains the fold change value of transcripts that are found only in susceptible JG62 plants whereas red line demonstrates the expression values of transcripts found exclusively in resistant WR315 plants. The region where red and blue lines overlap with one another explains transcripts that are found in both JG62 and WR315 plants. Green line independently explains the transcripts that are found to be induced in JG62 and WR315 plants only after infection. All the transcripts undulate significantly but the transcripts that are found to be induced only after infection exhibit maximum expressional values. The surface distribution of the transcripts also exhibited defense, metabolism, signaling and ROS regulations with varied expressional values (Fig 4; S8 Table, S9 Table).

### Gene ontology and functional classification

**Fig 5:**
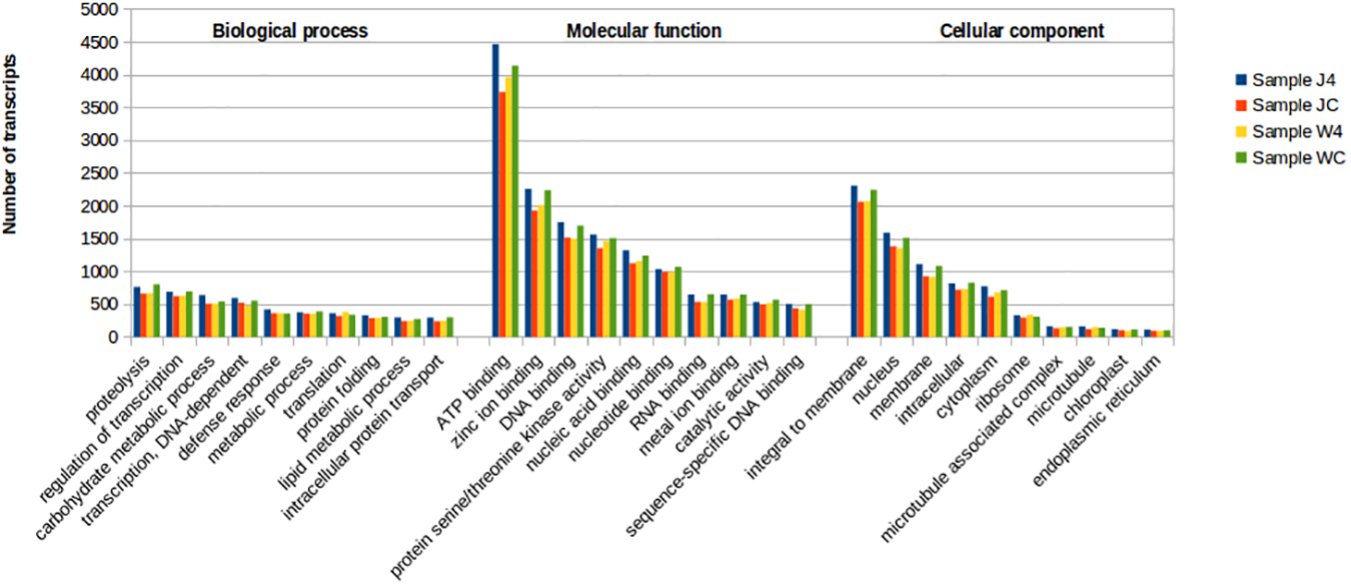
Gene ontology based analyses and functional clustering of total transcripts. Gene ontology study display varying distribution of transcripts across biological process, molecular function and cellular components.

Clustering of total annotated transcripts based on gene ontology showed varying distribution of transcripts across biological process, molecular function and cellular components (Fig 5). Amongst several biological processes, proteolysis, regulation of DNA dependent and independent transcription, carbohydrate metabolism showed maximum number of transcripts for all the four samples. Direct defense response was found to be controlled by comparatively less number of transcripts. Amongst the transcripts regulating molecular function, transcripts normalizing ATP binding were found to be the highest. Other transcripts related to binding were also found in fairly large numbers. Presence of relatively more number of transcripts related to membrane, nucleus and cytoplasm were also found (Fig 5).

**Fig 6:**
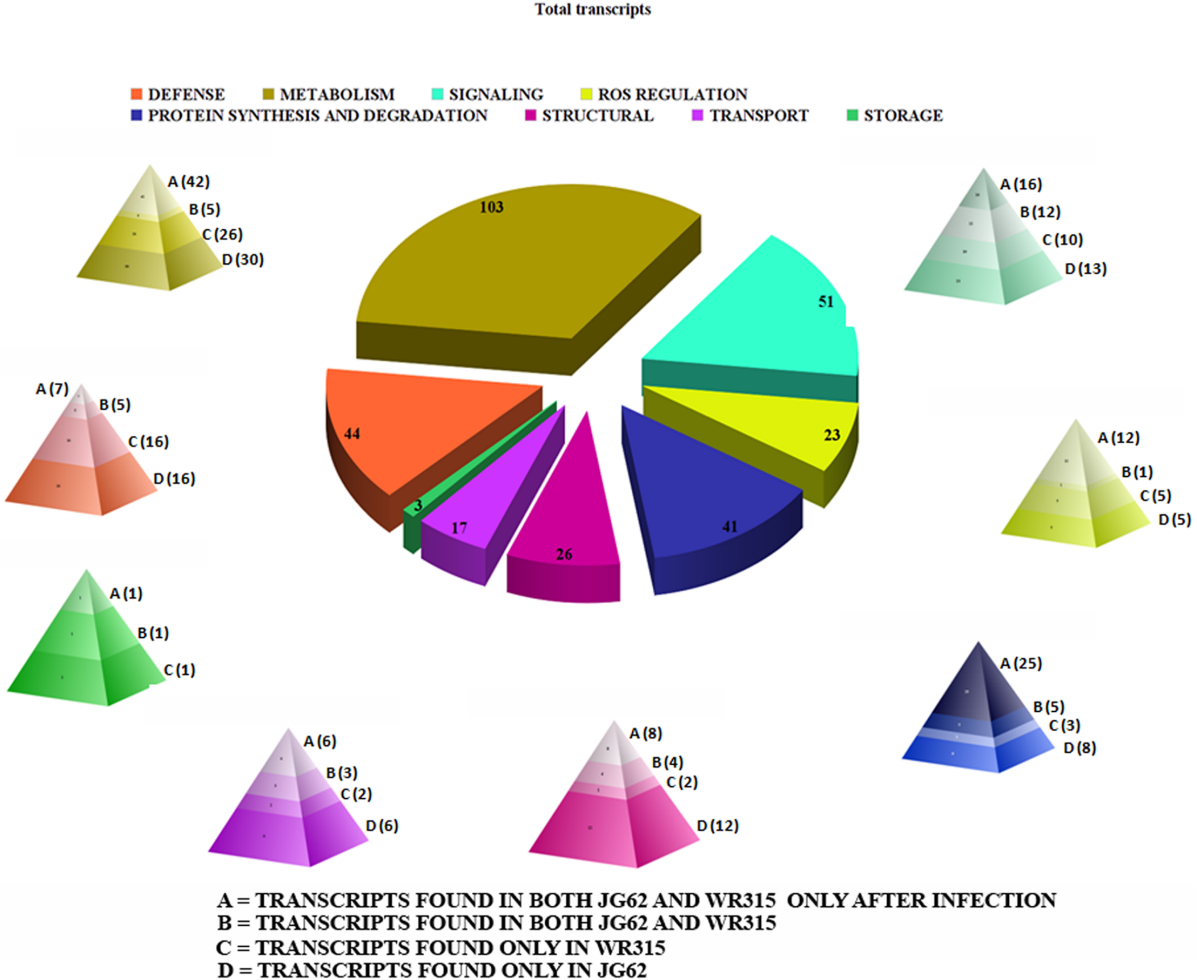
Functional clustering of total transcript with fragmented pie chart using Chart Tool software package. Individual pie fragments are distributed into gene pyramids having four zones. **A**. represents transcripts found in both JG62 and WR315 only after infection; **B**. represents transcripts found in both uninduced JG62 and WR315, **C**. represents transcripts found only in WR315 after infection and **D.** represents transcripts found only in JG62 after infection.

Functional categorization demonstrates that among eight distinct categories the highest number of transcripts belongs to the metabolism group. Signaling and defense related transcripts are the other two major groups that are found to be induced after infection. Protein synthesis and degradation events were also found to be induced [20]. Besides, structural components, storage and transport related transcripts were also induced (Fig 6. S8 Table).

### qRT-PCR analyses of representative genes

**Fig 7:**
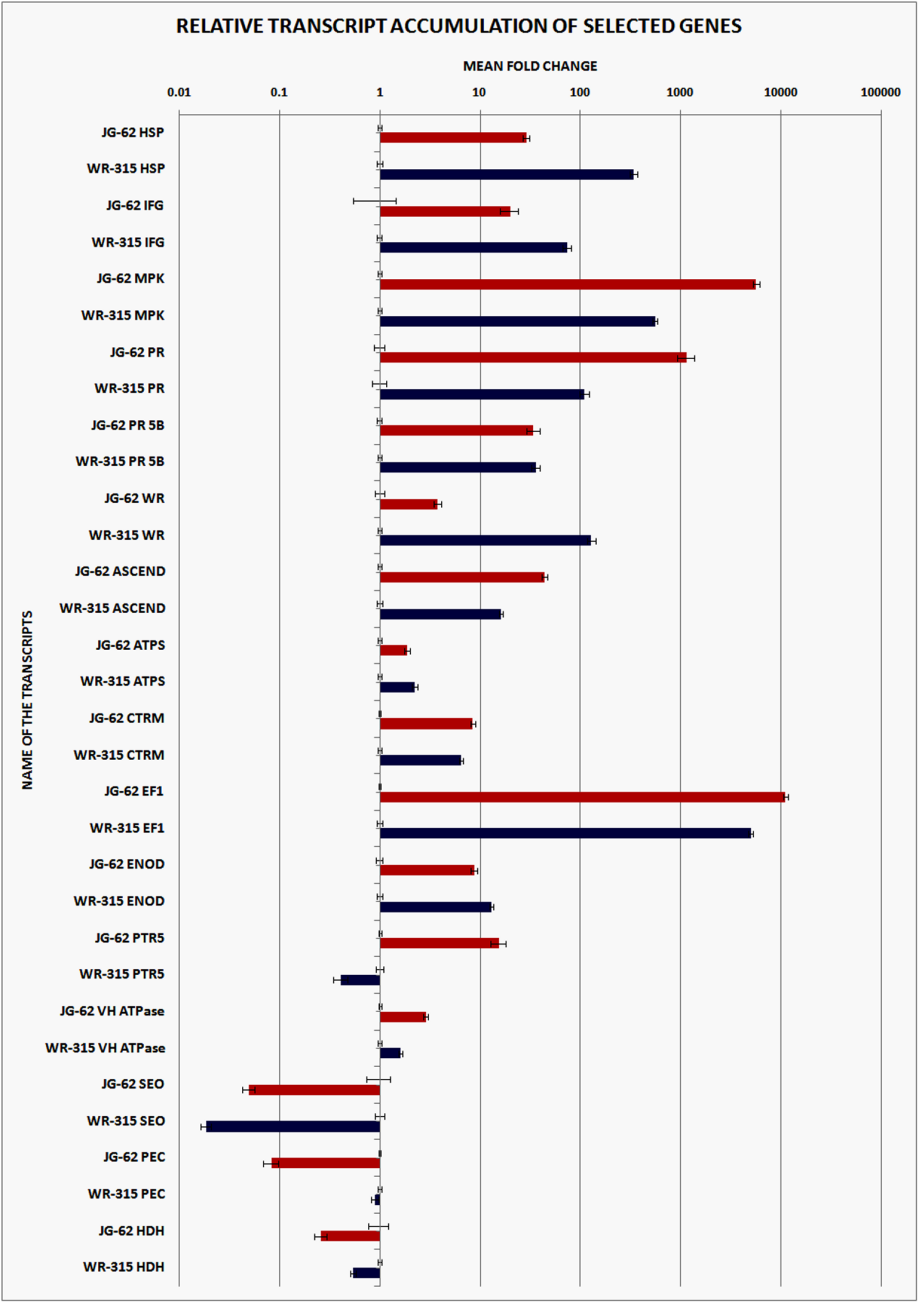
Quantitative realtime- PCR analyses of representative genes. Comparative transcript accumulation between susceptible (JG62) and resistant (WR315) chickpea genotypes on Foc1 infection were shown in the graph. JG62 represented by red color bars and WR315 represented by blue color bars. The genes selected are HSP (Heat shock protein), IFG ((Iso) flavonoid glycosyltransferase), MPK (Mitogen-activated protein kinase 2), PR (Pathogenesis Related protein), PR5B (Thaumatin-like protein PR-5b (Fragment)), WR (WRKY transcription factor), ASCEND (Acidic endochitinase), ATPS (ATPase alpha/beta chain), CTRM (Cytochrome P450 monooxygenase), EF1(Elongation factor 1-alpha), ENOD (Early nodulin), PTR5(Peptide transporter PTR5), VH ATPase (V-H(+)-ATPase subunit A), SEO (Sieve element occlusion c), PEC (Phosphoenolpyruvate carboxylase), HDH (Histidinol dehydrogenase).

Most of the selected transcripts showed up regulation in both JG62 and WR315 plants except SEO, PEC and HDH. Among the up regulated components of the transcripts EF1, MPK and PR1 displayed highest expressional induction and all the three transcripts show greater induction in susceptible plants as compared to the resistant plants. Besides, PR5B expression was found to be largely comparable in both JG62 and WR315 plants. In case of ASCEND the induction in JG62 is more than that of WR315 plants. Rest of the positively induced transcripts e.g. HSP, IFG, WR and ENOD the expression level of WR315 was more than that of JG62, whereas, other transcripts marginally varied among themselves. Contrarily, PTR5 induced in JG62 but the expression was dramatically reduced in WR315 plants. Among the down regulated transcripts except SEO, expression of other transcripts (PEC, HDH) were significantly low in case of susceptible (JG62) plants than that of resistant (WR315) plants (Fig 7).

### Network analyses of differentially expressed functional classes

**Fig 8:**
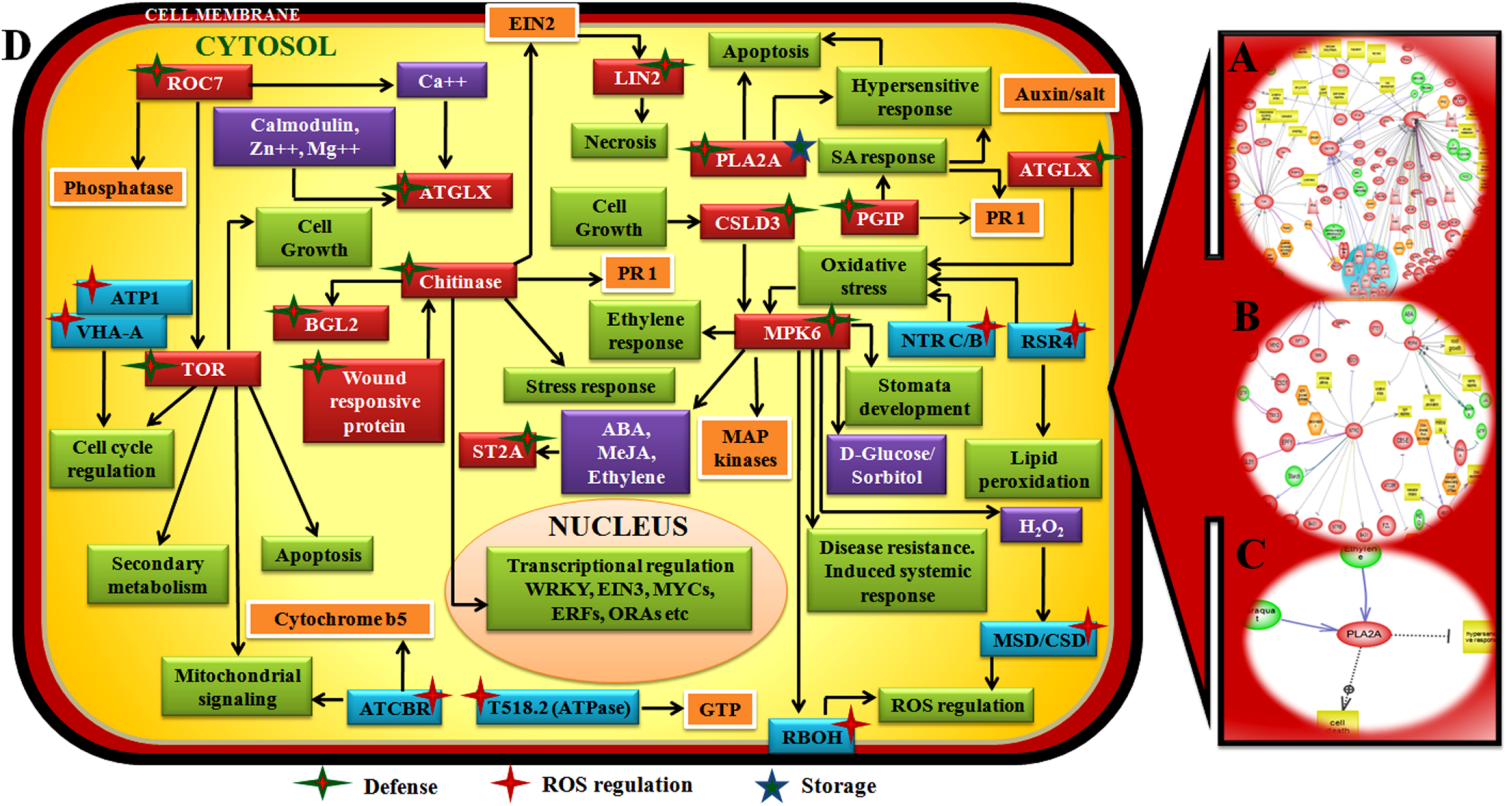
Network showing interaction between defense related components, ROS regulatory components and storage related components in chickpea during Foc1 attack. **A.** Network generated by pathway studio software showing interaction between defense related components. **B.** Network generated by pathway studio software showing interaction between ROS regulatory components. **C.**Network generated by pathway studio software showing interaction between storage related component. **D.** Manual integrated network showing interaction and interconnections between defense related components (red box, green bordered red star) ROS regulatory components (blue box, red star) and storage related component (black star). Defense components TOR(Serine threonine protein kinase), ROC (Peptidylprolylcis trans isomerase), BGL2 (Beta glucosidase), ATGLX (Lactolylglutathionelyase), ST2A (Sulfotransferase), MPK6 (MAP kinase), CSLD3 (Cellulose synthase), PGIP (Poly galactouronase inhibiting protein), LIN2 (Coproporphirinogen III oxidase), Storage component PLA2A (Patatin like protein), ROS related component ATP1(V type ATPase), VHA-A (V type ATPase), ATCBR(Cytochrome b5 reductase), RBOH (Respiratory burst oxidase), CSD/MSD (Manganese or copper superoxide dismutase), RSR4 (Reduced sugar response 4), NTRC/B (NTRC/ NADPH-dependent thioredoxinreductase C/B).

Network analyses was performed with only those set of TAIR protein homologues that showed interactions with at least a single neighbor while rest of the proteins showing no interconnections with relatives were eliminated. Interaction map showed the location of several defense responsive components such as TOR (Serine threonine protein kinase), ROC (Peptidylprolylcis trans isomerase), BGL2 (Beta glucosidase), ATGLX (Lactolylglutathionelyase), ST2A (Sulfotransferase), MPK6 (MAP kinase), CSLD3 (Cellulose synthase), PGIP (Poly galactouronase inhibiting protein) and LIN2 (Coproporphirinogen III oxidase). Storage component PLA2A (Patatin like protein) and ROS related component ATP1 (V type ATPase), VHA-A (V type ATPase), ATCBR (Cytochrome b5 reductase), RBOH (Respiratory burst oxidase), CSD/MSD (Manganese or copper superoxide dismutase), RSR4 (Reduced sugar response 4), NTRC/B (NTRC/ NADPH-dependent thioredoxinreductase C/B) were also found (Table 1, Fig 8, S1 File).

**Table 1:** List of important differentially regulated transcripts based on their involvement in functional networking.

**A. Down regulated transcripts in JG62 plants after infection**	
**Name of the transcripts**	**log2 Fold induction**
Adenosine 5'-phosphosulfate reductase	**-3.017951144**
R2R3-MYB transcription factor LjPAP	**-3.786094817**
Aquaporin TIP1-1	**-3.356983722**
Sulfotransferase domain	**-3.4675374**
Cellulose synthase	**-3.442153704**
Beta-galactosidase (EC 3.2.1.23)	**-3.185449452**
Sucrose transport protein SUF1	**-4.036027466**
Early light inducible protein	**-6.099722141**
Fasciclin-like arabinogalactan protein	**-3.067840745**
Lactolylglutathione lyase	**-5.467189025**
Lipoxygenase (EC 1.13.11.-) (Fragment)	**-3.221480032**
Aquaporin TIP2-1	**-4.0158952**
**B. Down regulated transcripts in WR 315 plants after infection**	
Chlorophyll a/b binding protein	**-3.765691033**
Phosphoenolpyruvate carboxylase (EC 4.1.1.31)	**-5.596801252**
Chlorophyll a-b binding protein	**-4.890841715**
Nitrate transporter	**-5.091016097**
**C. Up regulated transcripts in JG62 plants after infection**	
Tubulin alpha-1 chain	**7.75686448**
Serine hydroxymethyltransferase (EC 2.1.2.1)	**7.426219168**
Alcohol dehydrogenase	**3.314887146**
Peptidyl-prolyl cis-trans isomerase (EC 5.2.1.8)	**7.923198281**
NifU-like protein	**7.900899614**
26S protease regulatory subunit 6A-like protein	**8.724217246**
Class I chitinase (EC 3.2.1.14)	**3.982807189**
Mitogen-activated protein kinase 2	**10.67250221**
Serine/threonine protein kinase atr	**4.103109021**
Regulator of nonsense transcripts-like protein	**10.07531201**
S-adenosylmethionine synthase (EC 2.5.1.6)	**8.487359463**
Coproporphyrinogen-III oxidase	**8.739377054**
Heat shock protein	**9.531572169**
ATP synthase subunit beta (EC 3.6.3.14)	**9.3492571865**
70 kDa heat shock cognate protein 1	**8.805691308**
Malate synthase (EC 2.3.3.9)	**7.872532695**
1-aminocyclopropane-1-carboxylate oxidase	**5.833036161**
V-H(+)-ATPase subunit A	**9.183648865**
KRR1 small subunit processome component (KRR-R motif-containing protein 1)	**8.449451475**
Trans-cinnamate 4-monooxygenase	**4.57209532**
DNA polymerase (EC 2.7.7.7)	**8.178096472**
Succinate dehydrogenase	**10.44406397**
ATP synthase subunit alpha	**8.640020898**
Histone deacetylase (EC 3.5.1.98)	**7.667227268**
Ubiquitin fusion protein	**7.769226981**
Lipoyl synthase 1, mitochondrial (EC 2.8.1.8) (Lipoate synthase 1) (LS 1) (Lip-syn 1) (Lipoic acid synthase 1)	**9.170659883**
**D. Up regulated transcripts in WR 315 plants after infection**	
Elongation factor 1-alpha (EF-1-alpha)	**12.61190464**
Putative wound-induced protein	**5.028077411**
K(+)/H(+) antiporter (Sodium/hydrogen exchanger)	**4.434440792**
Patatin-like protein	**7.897366814**
FACT complex subunit SSRP1	**8.875273043**
Beta-fructofuranosidase, cell wall isozyme	**4.741711443**
MYB transcription factor	**6.270584137**
Glutamate dehydrogenase	**6.333547636**
WRKY transcription factor WRKY109669	**4.722667518**
Asparagine synthetase (EC 6.3.5.4)	**9.705348041**
Cellulose synthase-like protein	**4.229319912**
Ethylene responsive transcription factor 2b	**3.699398775**
CRT/DRE binding factor 4	**3.878466361**
Polygalacturonase inhibiting protein	**5.487985494**
Putative respiratory burst oxidase-like protein A	**3.800602718**

**Fig 9:**
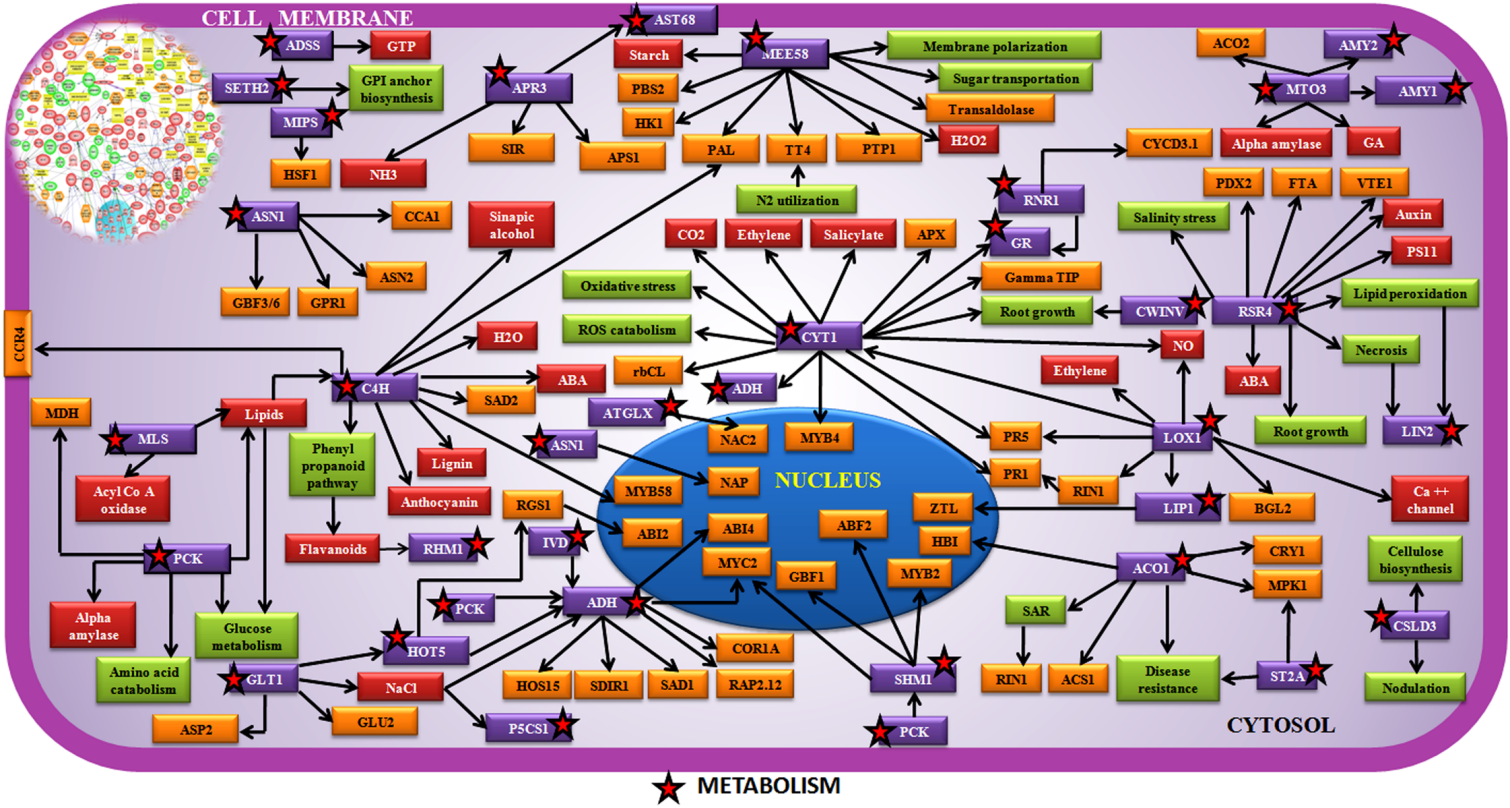
Network showing interaction between metabolic components in chickpea during Foc1 attack. **Inset:** Network generated by pathway studio software showing interaction between metabolism related components. Manual integrated network showing interaction and interconnections between metabolism related components (violet box, black bordered red star). MEE58(S-adenosyl-L-homocysteine hydrolase), CYT1(Cytokinesis defective 1), GLT1(Glucose transporter 1), MTO3 (Methionine over-accumulator 3), APR3(APS reductase 3), C4H(Cinnamate 4-hydroxylase), LOX1(Lipoxygenase 1), PCK1(Phosphoenolpyruvate carboxykinase), CWINV1(Cell wall invertase 1), ADH1(Alcohol dehydrogenase), ACO1(ACC oxidase 1), MLS (Malate synthase), ASN1(Glutamine-dependent asparagine synthase 1), RNR1(Ribonucleotide reductase 1), HOT5 (Sensitive to hot temperatures 5), SHM1(Serine hydroxymethyltransferase 1), FDH (Formate dehydrogenase), P5CS1(Delta1-pyrroline-5-carboxylate synthase 1), LIP1(Lipase 1), SDH1(Succinate dehydrogenase), RHM1(Rhamnose biosynthesis 1), AMY1 (Alpha-amylase-like), SBE2.2 (Starch branching enzyme 2.2), MTLPD2 (Lipoamide dehydrogenase 2), MIPS2 (Myo-inositol-1-phosphate synthase 2), IPP2 (Isopentenyl pyrophosphate:dimethylallyl pyrophosphate isomerase 2), GDH3 (glutamate dehydrogenase 3),TIM (Glyceraldehyde dehydrogenase phosphate), IVD (Isovaleryl-coa-dehydrogenase), PMDH1 (Peroxisomal nad-malate dehydrogenase 1), ADSS (Adenylosuccinate synthase), GR (Glutathione reductase), SUS4 (Sucrose synthase 4).

**Fig 10:**
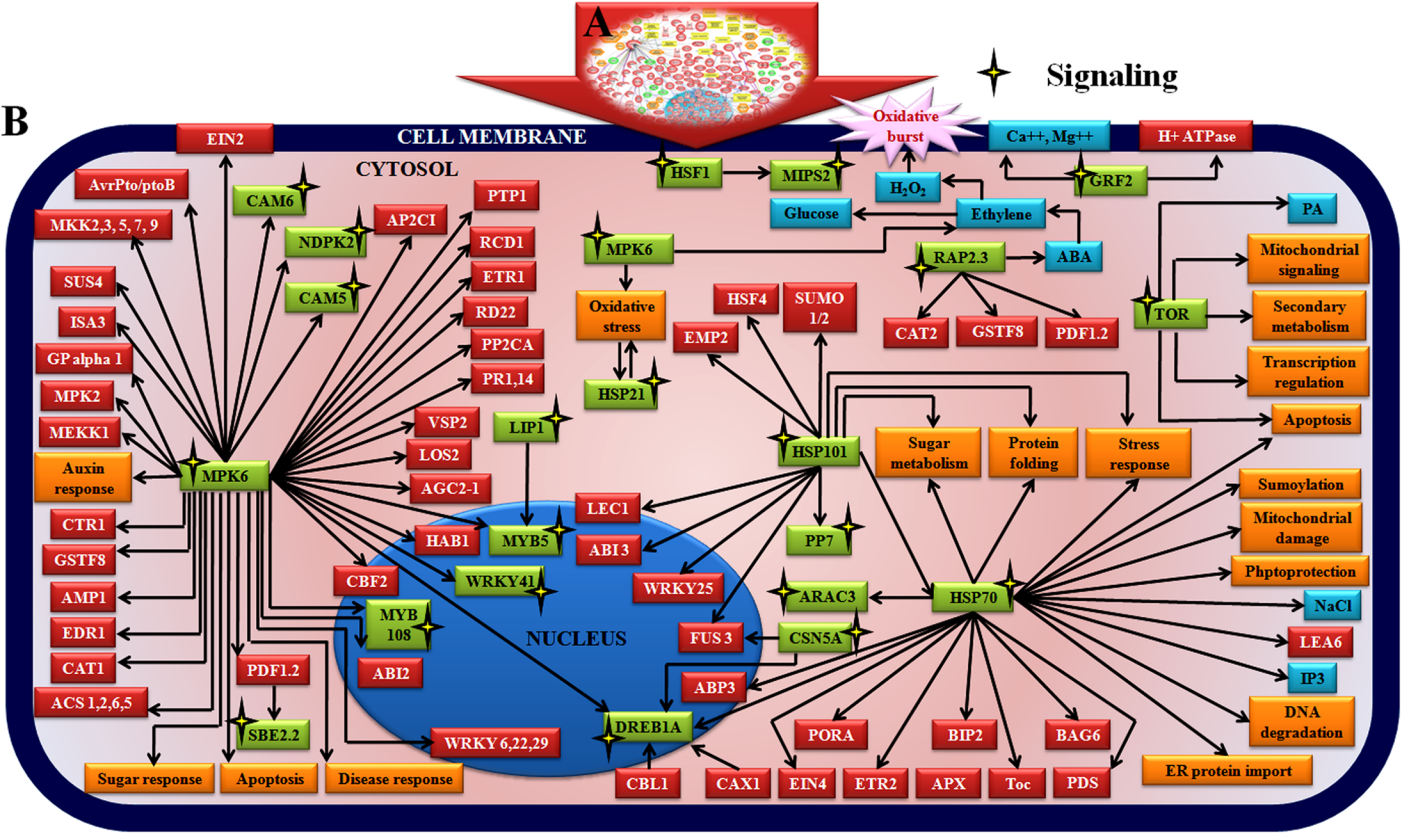
Network showing interaction between signaling components in chickpea during Foc1 attack. **Inset:** Network generated by pathway studio software showing interaction between signaling regulatory components. Manual integrated network showing interaction and interconnections between signaling related components (green box, black bordered yellow star). CAM5 (Calmodulin protein 5), CAM7 (Calmodulin protein 7), HSF1 (Heat shock factor 1), HSP101 (Heat shock protein 101), HSP70 (Heat shock protein 70), GRF2 (14-3-3 G box binding protein), MYB5 (MYB transcription factor), MYB108 (MYB transcription factor), WRKY41 (WRKY transcription factor), RAP2.3 (Ethylene responsive transcription factor 2b), DREB1A (CRT/DRE binding factor 4), NDPK2 (Nucleoside diphosphate kinase), CSN5A (COP9 Signalosome 5A), ARAC3 (GTPase).

Large number of metabolic components like MEE58(S-adenosyl-L-homocysteine hydrolase), CYT1(Cytokinesis defective 1), GLT1(Glucose transporter 1), MTO3 (Methionine over-accumulator 3), APR3(APS reductase 3), C4H(Cinnamate 4-hydroxylase), LOX1(Lipoxygenase 1), PCK1(Phosphoenolpyruvate carboxykinase), CWINV1(Cell wall invertase 1), ADH1(Alcohol dehydrogenase), ACO1(ACC oxidase 1), MLS (Malate synthase), ASN1(Glutamine-dependent asparagine synthase 1), RNR1(Ribonucleotide reductase 1), HOT5 (Sensitive to hot temperatures 5), SHM1(Serine hydroxymethyltransferase 1), FDH (Formate dehydrogenase), P5CS1(Delta1-pyrroline-5-carboxylate synthase 1), LIP1(Lipase 1), SDH1(Succinate dehydrogenase), RHM1(Rhamnose biosynthesis 1), AMY1 (Alpha-amylase-like), SBE2.2 (Starch branching enzyme 2.2), MTLPD2 (Lipoamide dehydrogenase 2), MIPS2 (Myo-inositol-1-phosphate synthase 2), IPP2 (Isopentenyl pyrophosphate:dimethylallyl pyrophosphate isomerase 2), GDH3 (Glutamate dehydrogenase 3),TIM (Glyceraldehyde dehydrogenase phosphate), IVD (Isovaleryl-coa-dehydrogenase), PMDH1 (Peroxisomal nad-malate dehydrogenase 1), ADSS (Adenylosuccinate synthase), GR (Glutathione reductase), SUS4 (Sucrose synthase 4) were found to interconnect in the metabolic regulatory pathway (Table 1, Fig 9, S1 File). Signal regulating molecules such as CAM5 (Calmodulin protein 5), CAM7 (Calmodulin protein 7), HSF1 (Heat shock factor 1), HSP101 (Heat shock protein 101), HSP70 (Heat shock protein 70), GRF2 (14-3-3 G box binding protein), MYB5 (MYB transcription factor), MYB108 (MYB transcription factor), WRKY41 (WRKY transcription factor), RAP2.3 (Ethylene responsive transcription factor 2b), DREB1A (CRT/DRE binding factor 4), NDPK2 (Nucleoside diphosphate kinase), CSN5A (COP9 Signalosome 5A), ARAC3 (GTPase) were found to be located in the network (Table 1, Fig 10, S1 File).

**Fig 11:**
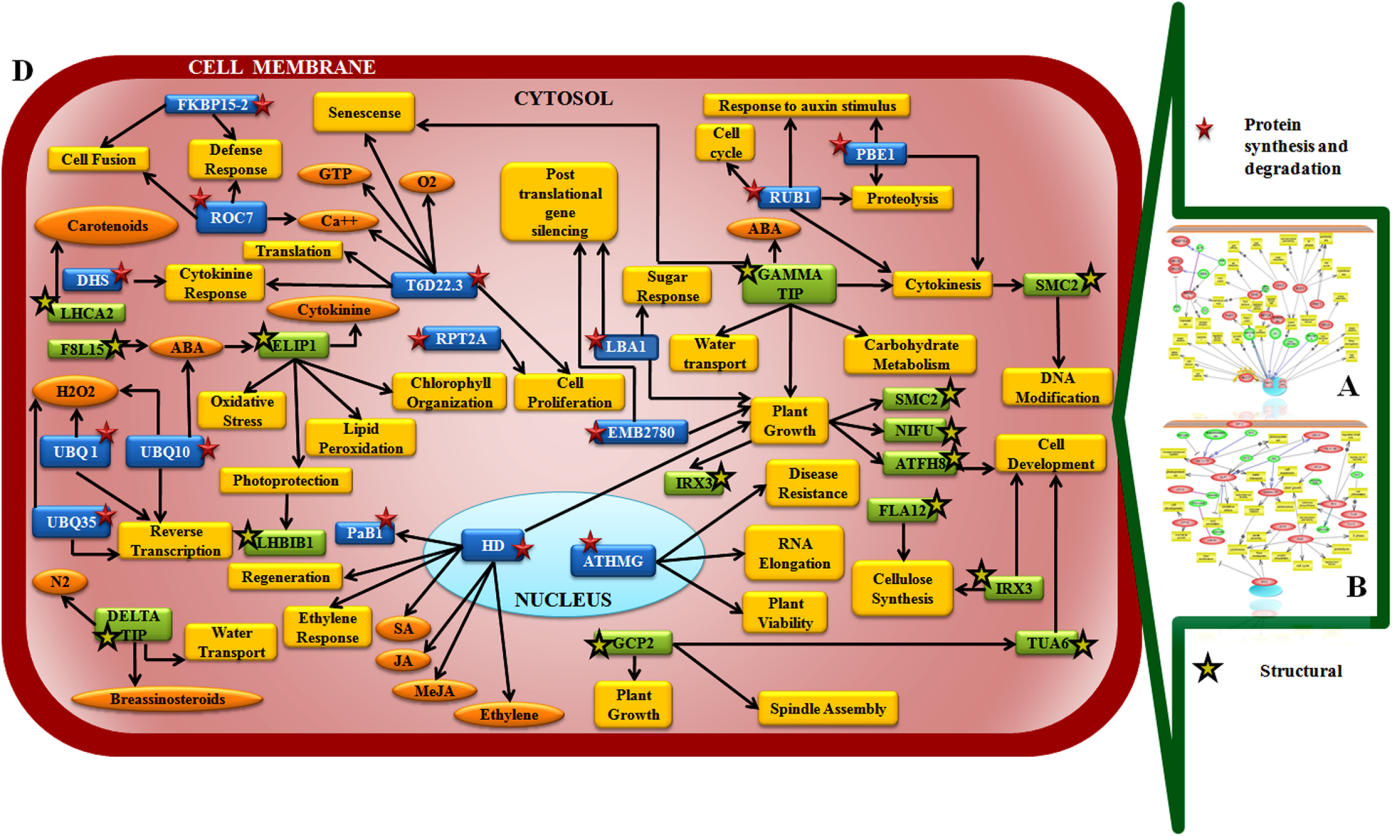
Network showing interaction between protein synthesis and degradation related components and structural components in chickpea during Foc1 attack. **A.** Network generated by pathway studio software showing interaction between protein synthesis and degradation related components. **B**. Network generated by pathway studio software showing interaction between structural components. **C.** Manual integrated network showing interaction and interconnections between protein synthesis and degradation related components (green box, black bordered yellow star) and structural components (blue boxes red stars). Protein synthesis and degradation related components FKBP15-2 (Peptidyl-prolyl cis-trans isomerase), UBQ1(Ubiquitin 1), UBQ10 (Ubiquitin 10), UBQ35 (Ubiquitin 35), PaB1(Proteasome subunit), HD (Histone deacetylase), RPT2A (Proteasome component), T6D22.3 (Elongation factor), EMB2780 (DNA polymerase), LBA1(Regulator of nonsense transcript like protein), RUB1 (Ubiquitin), PBE1(Proteasome components), ATHMG (FACT complex subunit SSRP1); structural components LHB1B1(Chlorophyll a/b binding protein), LHCA2 (Chlorophyll a/b binding protein), Delta TIP (Delta Tonoplanst intrinsic protein), Gamma TIP (Gamma Tonoplanst intrinsic protein), ELIP1(Early light inducible protein), GCP2(Tubulin gamma chain), FLA12 (Fasciclin like arabinogalactan protein), IRX3 (Cellulose synthase), ATFH8 (Formin like protein), NFU4 (NifU like protein), TUA6 (Tubulin alpha chain), SMC2 (Structural maintenance of chromosome), F8L15.150 (KRR motif containing protein 1).

Protein synthesis and degradation related components FKBP15-2 (Peptidyl-prolyl cis-trans isomerase), UBQ1(Ubiquitin 1), UBQ10 (Ubiquitin 10), UBQ35 (Ubiquitin 35), PaB1(Proteasome subunit), HD (Histone deacetylase), RPT2A (Proteasome component), T6D22.3 (Elongation factor), EMB2780 (DNA polymerase), LBA1(Regulator of nonsense transcript like protein), RUB1 (Ubiquitin), PBE1(Proteasome components), ATHMG (FACT complex subunit SSRP1) were mapped in the interaction pathway. Besides several structural components like LHB1B1(Chlorophyll a/b binding protein), LHCA2 (Chlorophyll a/b binding protein), Delta TIP (Delta Tonoplanst intrinsic protein), Gamma TIP (Gamma Tonoplanst intrinsic protein), ELIP1(Early light inducible protein), GCP2(Tubulin gamma chain), FLA12 (Fasciclin like arabinogalactan protein), IRX3 (Cellulose synthase), ATFH8 (Formin like protein), NFU4 (NifU like protein), TUA6 (Tubulin alpha chain), SMC2 (Structural maintenance of chromosome), F8L15.150 (KRR motif containing protein 1) were found (Table 1, Fig 11, S1 File).

**Fig 12:**
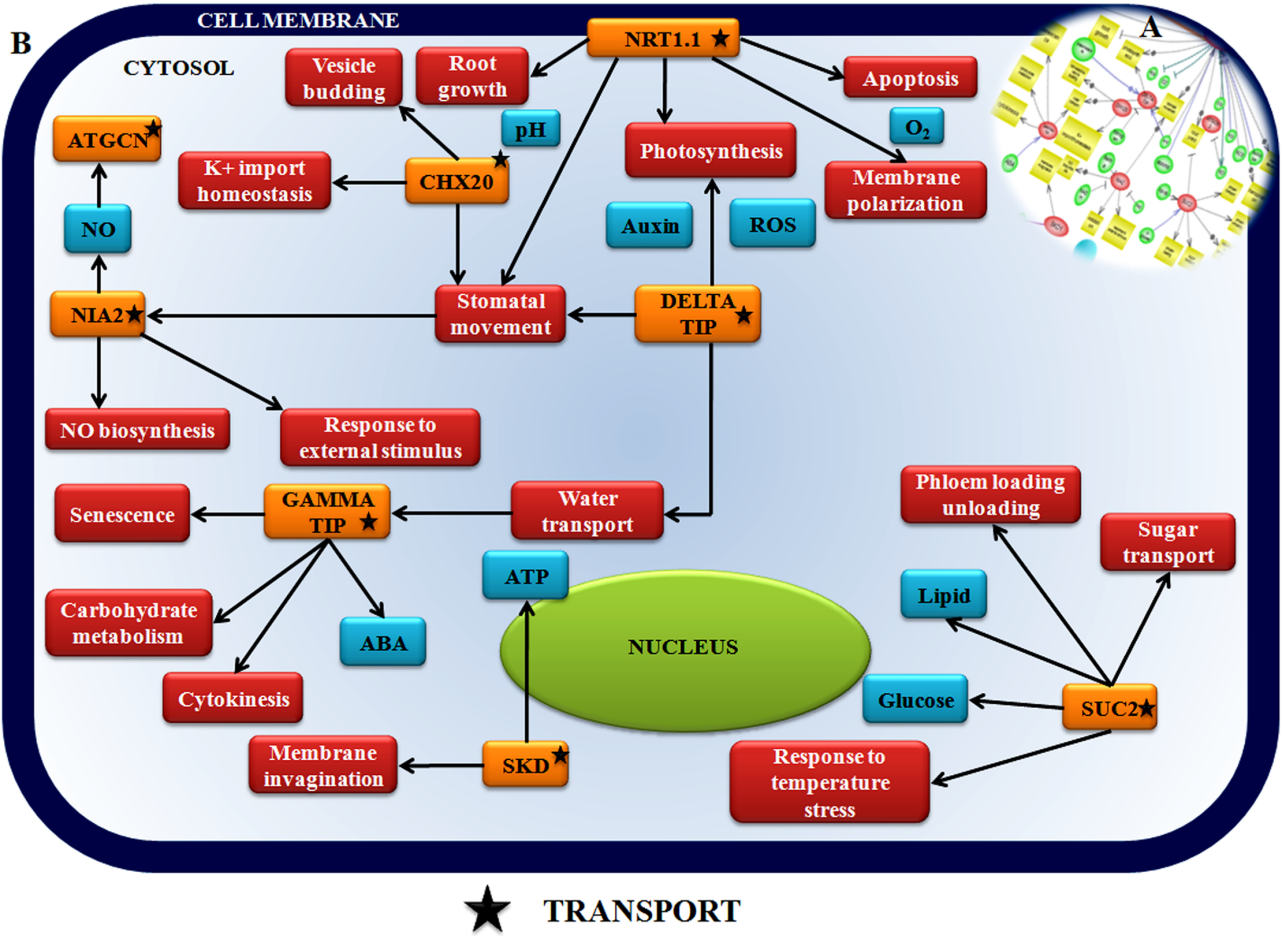
Network showing interaction between transport regulatory components in chickpea during Foc1 attack. **Inset:** Network generated by pathway studio software showing interaction between transport regulatory components. Manual integrated network showing interaction and interconnections between signaling related components (orange box, black star). CHX20 (K+/N+ antiporter), SKD (Vacuolar sorting protein), NRT (Nitrate transporter), ATGCN (ABC transporter family protein), SUC 2 (Sugar transporter).

Transport controlling components such as CHX20 (K+/N+ antiporter), SKD (Vacuolar sorting protein), NRT (Nitrate transporter), ATGCN (ABC transporter family protein), SUC 2 (Sugar transporter) were found to interact in the interaction pathway (Table 1, Fig 12, S1 File).

Amongst the several components mentioned above, PLA2 showed overlapping role in storage and defense. Besides, ATGLX1, ST2A, LIN2 and CSLD3, served as regulators of direct defense, as well as acted in regulating host metabolic activities. TOR, MIPS and LIP1 showed overlapping roles in regulating defense, metabolism as well as signaling, Gamma-TIP a structural component regulated metabolism and transport. RUB also being a structural component regulated protein synthesis and degradation.

## Discussion

Plants use above ground green tissues to convert solar energy to biological energy that directly or indirectly sustains all life forms on earth. But, the essentiality of roots in fuelling this life sustenance process remains mostly ill noticed [21]. In addition, damages and yield losses caused by perpetuate soil borne pathogens are also underestimated. In fact prevention of major crop losses due to root pathogens, known in general to be even more difficult to control than diseases of leaves and fruits [21]. In the present case study of chickpea–*Fusarium* interaction attempts have been made to develop an understanding of how roots trigger defense signaling cascades and tackle the scuffle with its invading pathogen using RNA-seq approach. This study reports a large number of transcripts with low non ATGC characters marking the library to be of good quality. Radar plot analyses showing maximum expressional values of defense, metabolism, signaling and ROS regulation related transcripts projected them as key regulators of defense in chickpea-Foc1 interaction. GO based analyses showed comparatively less number of transcripts related to direct defense as compared to transcripts regulating transcription, carbohydrate metabolism and proteolysis suggesting that imparting defense to a system is a complex phenomenon, probably controlled by multidimensional regulatory mechanism. Besides large number of transcripts regulating ATP binding suggested a constant need of energy to meet up internal as well as external requirements [22]. Abundance of binding related transcripts indicated its importance in imparting defense. Site specific interaction was predicted by the presence of relatively more number of transcripts related to membrane, nucleus and cytoplasm [22]. The study of transcript accumulation through quantitative realtime PCR exhibits similar pattern of expression as observed by DESeq analyses of NGS. Some genes deviate slightly from DESeq data as an obvious phenomenon as DESeq relies on base mean fluorescence value but qRT-PCR relies on absolute transcript accumulation specifically targeted by gene specific primers. Interaction analyses showing location and interconnection of different transcripts belonging to diverse biological classes indicated the presence of an intricate defense responsive network in chickpea that presumably controlled the pathogenic outcome against Foc1.

### Defense, ROS and storage related genes interact in defense responsive pathway

ROCs (Peptidyl proplyl cis trans isomerase), a class of plant immunophilins/cyclophilins are reported to catalyze the activation of several pathogen effectors by bringing about their conformational alterations and triggering R gene mediated host defense response [23]. Soybean cyclophilin was reported to induce oxidative stress response and cell death at the site of infection by activating the effector of *Phytopthora sojae* [24]. In present study homologous to ROC7 was found to be induced upon infection in susceptible JG62 plants while down regulated in WR315 plants. Such induction strongly predicts the involvement of a pathogen effector in the present study, which is unidentified till date. TOR (Target of rapamycin) represents a group of conserved serine threonine kinases which belong to the phosphatidylinositol kinase related kinases (PIKK) are catalyzed by plant cyclophilins [25]. TOR proteins are regulators of both anabolic and catabolic processes such as cell growth, cell cycle regulation, secondary metabolism, mitochondrial signaling and apoptosis, with regulating autophagy and cell wall structure and development being their primary roles [26]. In present study, TOR was found to be induced in JG62 upon infection suggesting changes in cell wall integrity during pathogen invasion. Besides induction of autophagy, reported to induce cell death in susceptible plants was also previously reported [16]. Conversely, the induction of TOR in resistant plants indicated towards regulating defense signaling by probably modulating secondary metabolism. BGL (Beta 1,3 glucanase) protein expression is known to be increased significantly upon infection by diverse group of pathogen [27]. They are also known to function as plasmodesmatal gatekeepers mediating intercellular communication [28]. In present study BGL was found to be upregulated in both JG62 and WR315 suggesting a common mechanism to be operational during compatible and incompatible interaction with Foc1 with comparative higher concentrations of BGL in resistant plants indicating towards a resistance response probably controlled by quantitative thresholds. On the other hand Oschib (Chitinase), a homologue of chitinase class of pathogenesis related proteins was found to be significantly increased in resistant WR315 plants suggesting its profound role in plant defense against Foc1 attack. Chitinases are defined as important arsenal of plants against fungal pathogens and molecular targets of selection in plant-pathogen coevolution [29]. They act in combination with BGLs causing hydrolysis of hyphal tips [29]. Interestingly, induction of chitinase in resistant plants not only predicts its emergence as a product of selective coevolution but also indicates about the existence of an unconventional systemic response during infection by Foc1 that is known to cause localized responses. Besides, chitinases are known to regulate expression of PR1 and wound inducible protein T9A4.6 [30]. ATGLXs (Lactolylglutathione lyase) also named as glyoxalase I are known to detoxify methylglyoxal formed during ROS generation under stressful conditions. Glyoxylase I in combination with ROS scavenging enzymes strikes a fine tune balance between the ROS generation and its detoxification [31]. In present study, down regulation of glyoxylase I in susceptible plants points out at a defunct antioxidant system and predicts the accumulation of cytotoxic methylglyoxals that cause protein and cell wall damage. STs (Sulfotransferases) catalyze the sulphation of wide range of substrates such as brassinosteroids, glucosinolates, secondary metabolites etc, majority of which contribute towards imparting host defense against harmful pathogens [32]. The downregulation of ST2A in susceptible plants suggests the low production of important sulphated intermediates that are meant to aid or boost host fitness against pathogenic upheaval. MPK6 (Mitogen activated protein kinase 6) is reported to act as both activator as well as repressor of defense in *Glycine max*, where the decisive role is determined by MKK4, the upstream factor of MPK6 [33]. MPK6 also regulates ethylene signaling, stomatal closure, systemic responses, oxidative stress and cell wall development [34, 35]. The upregulation of MPK6 in susceptible plants in the present study directs towards its negative role in defense as it probably gets hijacked by the pathogen that utilizes it to promote virulence. CSLD (Cellulose synthase) is known to help in glucan deposition and maintain cell wall integrity during pathogen invasion [36]. Induction of CSLD type 3 in resistant plants predicts its role in protecting cell wall from damage whereas susceptible plant probably fails to manage such devastations. PGIPs (Poly galactouronase inhibiting proteins) are known to inhibit the pectin depolymerizing activity of polygalactouronases (PG) secreted by a microbial pathogen and insects for initiating pathogenicity in host plants [37]. The PG-PGIP interaction not only controls defense outcome but also triggers downstream production of oligogalactouronides (OGs) that function as elicitors for activation of host defense signaling cascades involving expression of PR1and salicylic acid regulated responses [38]. The induction of PGIP in resistant plants put forth the importance of this component in modulating defense in the present case study. LIN2 (Coproporphyrinogen III oxidase) is the key enzyme of the tetrapyrrole biosynthetic pathway modulates apoptosis in *Arabidopsis thaliana* during powdery mildew disease [39]. The enhanced expression of LIN2 in susceptible plants indicates towards cell apoptosis. According to our previous studies apoptotic changes in vascular tissues is known to promote pathogen establishment [16]. PLA2A (Patatin like protein) are vacuolar storage proteins known to have regulatory functions in defense signaling through jasmonic acid or oxylipin accumulation [40]. They promote resistance to obligate pathogen cucumber mosaic virus by inducing late accumulation of oxylipins that limit HR spread [41]. Interestingly, accumulation of PLA2A in resistant plants foretell the presence of similar mechanism that somehow restricts pathogen cataclysm by shunting hypersensitive responses and cell death at host’s primary solute conducting tissue, the vascular bundle (Fig 8, S8 Table). The generation of ROS is the earliest temporal event following pathogen perception. They not only function as executioners of cell death but act as signaling molecules by triggering downstream defense. RBOH (Respiratory burst oxidase) encodes key enzyme subunit of plant NADPH oxidase that regulates ROS generation and channelizes them as signaling molecules with MPK6 acting as coregulator [42]. In present study upregulation of RBOH in the resistant plants supports its regulatory activity as signal generator by controlling ROS levels to optimal thresholds. ATPases are primary pumps that stabilize potentials across cellular membranes and in the process maintains cellular homeostasis [43]. In present case study ATPases were found to be elevated in both compatible and incompatible interaction signifying the requirement of energy to sustain membrane potentials in both systems while facing pathogen encounters. *Arabidopsis thaliana* and *Oryza sativa* NTRs (Thioredoxin reductase) transfer reducing power to thioredoxin/peroxiredoxin system for scavenging ROS [44]. Additionally, SODs (Superoxide dismutase) serve as first line of defense against the detrimental effects of cellular ROS [45]. Upregulation of NTR B/C and Mn/CuSOD in resistant plants suggest the presence of an efficient antioxidant system that helps in resistance in chickpea against Foc1. RSR4 (Reduced sugar response 4) a mutant form of pyridoxine biosynthetic protein 1 (PDX1) controls vitamin B6 biosynthesis. Vitamin B6 eliminates oxidative stress while its phosphorylated forms participate in membrane maintenance by regulating lipid peroxidations. In present study higher accumulation of RSR4 indicates its role in promoting resistance by alleviating oxidative stress factors. ATCBR (*Arabidopsis thaliana* cytochrome b5 reductase) acts as important component of electron transport chain that regulates oxidoreductase activity and in the process regulates redox signaling [46]. Upregulation of CBR homologue in resistant plants also indicates towards its positive role in redox regulation (Table 1, Fig 8, S8 Table).

### Host metabolism modifies accordingly to combat pathogen devastations

The invariable tug-of-war between the metabolic systems of the interacting partners (plant-pathogen) decides the upshot of interaction. In present study several metabolic transcripts were found to be integrated in the defense regulatory network. MEE (S-adenosyl L-homocysteine hydrolase) maintains the methylation potentials in an organism. Downregulation of MEE was reported to lower H_2_O_2_ accumulation and promotes resistance in tomato against *Pseudomonas syringae* [47]. Upregulation of MEE in susceptible chickpea plants indicated towards accumulation of H_2_O_2_ that was reported to act as negative regulator of defense in chickpea-Foc1 interaction [16]. CYT1 (Cytokinesis deficient 1) is linked to N linked glycosylation that regulates cellulose biosynthesis. Studies on *Arabidopsis* suggest that the expression of CYT1 is associated with expression of PR genes and changes in ethylene and salicylic acid induction [48]. Upregulation of CYT1 in resistant plants post induction indicates towards its role in balancing ROS, repairing cell wall damages and regulating PR gene expression. Need based availability of soluble sugars are necessary for priming host against pathogen attack, where sugar transporters like GLT1 (Glucose transporter1) have profound role. They contribute as signal communicators right from the sink created at the site of fungal invasion to the plant interior. GLTs directly control sugar production that stimulate flavanoid production, PR gene expression and cell wall lignifications, all of which are thought to be protective measures needful for boosting host defense [49]. Thus, upregulation of GLT1 in resistant plants following infection emphasizes on its role in imparting resistance possibly by regulating one or all of the above features (Table 1, Fig 9, S8 Table).

MTO3 (Methionine over accumulator 3) is known to regulate polyamine and ethylene biosynthesis. Besides, MTO3 was reported to be co-expressed with ACO2 (ACC oxidase) during water stress in maize [50]. In present study upregulation of MTO3 in susceptible plants indicated induction of water stressed condition caused by Foc1 colonization. APR3 (APS reductase) tightly regulates sulphate assimilation and transport. Sulphate compounds in combination with glutathione ascorbate pathway components are known to detoxify ROS during stressed condition [51]. Down regulation of APR3 in susceptible plants suggested lack of proper sulphate transport and assimilation that was probably hijacked by the attacking Foc1. C4H (Cinnamate 4 hydrolase) is a cytochome P450 that catalyzes the synthesis of lignin, pigments and many defense molecules such as phenyl ammonia lyase (PAL), secondary metabolites, sinapic alcohol etc. [52]. Upregulation of C4H in susceptible plants in the present study suggests an attempt of the compatible plants to stimulate its self defense arsenal to combat pathogen attack which probably failed to reach the optimal levels. LOX1 (Lipoxygenase 1) directly regulates defense response by regulating expression of PR genes, ethylene, ABA levels and calcium channels [53]. Downregulation of LOX1 in susceptible plants points towards pathogen’s overpowering the host defense metabolism. PCK (Phosphoenolpyruvate carboxykinase) is known to regulate gluconeogenesis. Although, previous reports suggest PCK to be induced during bacterial and oomycetes pathogen invasion, but its role in plant immunity is unclear [54]. Downregulation of PCK in resistant plants directs towards reprogramming of self metabolism for efficient energy utilization as well as minimization during needful hours that is perhaps adopted as a protective measure to combat Foc1 encounter (Table 1, Fig 9, S8 Table).

CWINV1 (Cell wall invertase1) is believed to function as the key enzyme for reconstruction of damaged cell wall [55]. Upregulation of this invertase suggests its protective as well as repairing role during Foc1 infection. ADH (Alcohol dehydrogenase 1) is well studied as a primary metabolic enzyme with multifaceted functions, which can both accelerate or retard *in planta* pathogen establishment [56]. Interestingly, upregulation of ADH was noticed in susceptible plants that were probably reprogrammed by the attacking Foc1 to prop up self establishment within compatible host interior. ACO1 (ACC oxidase1) regulates ethylene biosynthesis and cell death [57]. Upregulation of ACO1 in susceptible plants presumably elicits cell death, which according to our previous studies was reported to lead to susceptibility against Foc1 [15]. ASN (Glutamine dependent asparagines synthase 1) regulates primary nitrogen metabolism that fuels myriad basic metabolic activities during pathogen infection [58]. In present study, upregulation of ASN in resistant plants indicates optimal energy flow essential to carry on basal metabolic functions and assist resistance against Foc1. RNR1 (Ribonucleotide reductase 1) is crucial for regulating transcriptional reprogramming and DNA repair during programmed cell death (PCD) [59]. Upregulation of RNR1 in resistant plants following infection with Foc1 suggests transcriptional regulation and repairing measures taken by the resistant plants to counter pathogenic devastations. SHMT1 (Serine hydroxyl methyl transferase 1) regulates ROS generation by controlling photorespiratory pathways [60]. Upregulation of SHMT1 in susceptible plants whether was an attempt of the host to protect self or an act of the pathogen that probably took hold of the host metabolism for self sustenance needs to be experimentally validated (Table 1, Fig 9, S8 Table).

AST68 (Sulphate transporter) is important component regulating drought stress via ABA dependent pathways. Sulphur acts in maintaining cellular redox balance and mitigates damage caused by ROS [61]. In present study downregulation of AST68 in susceptible plants point towards lack of efficient ROS detoxification system which the resistant plants presumably possess. ADSS (Adenylosuccinate synthase) plays important role in purine biosynthesis [62]. Interestingly, de novo purine biosynthesis was related to pathogen growth of blast fungus of rice [63]. Thus, in the present study dowregulation of ADSS in resistant plant following infection by Foc1 could be concluded as an effective measure undertaken by the resistant plants to prevent *in planta* growth of Foc1. SETH2 (Glucosyltransferase 2) regulates GPI anchor biosynthesis and cell wall protection [64]. In present study induction of SETH2 following infection in resistant plants predicts their role in cell wall protection. MIPS2 (Myo inositol phosphate synthase 2) produces signaling molecule inositol phosphate (IP). Besides, it was reported to be enhanced during nematode infection as well as to lead to increased H_2_O_2_ production in sweet potato [65]. In the present study decrease of MIPS2 in resistant plants probably emphasizes on its protective role by lowering H_2_O_2_ levels reported to be hazardous for chickpea during Foc1 attack (Fig 9, S8 Table). MLS (Malate synthase) is a unique enzyme of glyoxylate cycle which is regulated lipid metabolism. Previous reports state that MLS is necessary for *in planta* survival of bacteria and fungi [66]. Thus, in the present study upregulation of MLS in susceptible plants indicate the changes in the enzyme levels to be perhaps driven by Foc1. RHM1 (Rhamnose biosynthesis 1) controls the biosynthesis of rhamnose, is a cell wall pectic polysaccharide and component of secondary metabolism like flavanoids and glycoproteins [67]. In present study increased expression of RHM1 in resistant plants following Foc1 attack predicts production of secondary metabolites used for shielding the incompatible host from fungal damage. P5CS1 (Delta 1 pyrroline 5 carboxylate synthase 1) is reported to be strongly induced under pathogen attack and also known to modulate proline contents that act as osmoprotectant [68]. In the present study enhanced expression of P5CS1 suggests its osmoprotectant activity as Foc1 is known to kindle water starved condition inside the host by plugging of its vascular tissue. GR (Glutathione reductase) modulates redox homeostasis and RNR catalysis both of which are known to play fundamental roles in plant defense against diverse biotic and abiotic stress agents [69]. In the present study increment of GR post Foc1 attack emphasized on the need to maintain redox balance which was supported by previous studies [16]. LIP1 (Lipoyl synthase 1) is important for functioning of several essential multienzyme complexes such as pyruvate dehydrogenase, α ketoglutarate dehydrogenase and glycine decarboxylase [70]. Upregulation of LIP1 in susceptible plants marks fungal control over host metabolism. AMY1/2 (Alpha amylase1/2) controls starch degradation and the use of soluble sugars for protection and osmoregulation [71]. In the present study decrement of AMY1/2 post infection in resistant plants suggests as a control measure opted by the host by depriving the attacking pathogen of soluble sugars (Table 1, Fig 9, S8 Table).

### Signaling genes regulate the defense network

Effective temporal and spatial defense signals determine the fate of pathogen’s endeavor within the host. CAMs, the conserved calcium sensors, are reported to interact with 14-3-3, calcium dependent protein kinase 3 (CDPK3) and MPK6 simultaneously during *Pseudomonas syringae* infection that is known to regulate sphingolipid pathway dependent PCD in *Arabidopsis* and tomato [72]. In present study upregulation of CAM5 and CAM7 (isoforms of CAM) in resistant plants after infection suggests their regulatory role by probably circumventing the ill effects of PCD. Studies conducted on *Arabidopsis* reported that NDPK2 was upregulated along with H_2_O_2_ accumulation in roots which in turn activated MPK6 downstream indicating that MPK6 activation via NDPK2 was redox regulated [73]. Decreased expression of NDPK2 in resistant plants following Foc1 attack in the present study envisages its role in regulating resistance by lowering H_2_O_2_ accumulation in roots. MPK6 is also involved in ABA-induced expression of CAT1coupled with H_2_O_2_ production [74]. Thus, over expression of MPK6 in susceptible plants probably explains the role of pathogen in overpowering the host MAPK signaling module to produce H_2_O_2_ which ultimately harms the host. Such supposition is supported by reports on *Arabidopsis thaliana*, where bacterial suppressors AvrPto /AvrPtoB are reported to activate host MAPK signaling and promote susceptibility, while incompatible plants suppress MAPK to promote resistance [75] (Table 1, Fig 10, S8 Table).

WRKY41 is known to interact with pathogen effector proteins PopP2 and AvrRps4 in *Arabidopsis thaliana* [76]. In present study overexpression of WRKY41 homologue in resistant plants suggest its role in regulating resistance probably by interacting with Foc1 effector proteins which needs to be identified. MYB108 regulates wound induced cell death and accumulation of ROS [77]. In present study MYB108 was found to be upregulated in resistant plants post Foc1 attack suggesting its similar role in regulating wound induced cell death and promoting resistance. MYB5 regulates mucilage secretion during cell wall biosynthesis during different stages of development [78]. However, their role in regulating defense as found by their down regulation in susceptible plants in the present study, remains yet to be elucidated. DREB1A is reported as key regulator of drought tolerance using both ABA dependent as well as independent pathways [79]. In the present study Foc1 is known to induce water starved conditions which grossly mimics drought like conditions *in planta*. The induction of DREB1A homologue in resistant chickpea plants suggests its role in protecting the host from the ill effects of fungal induced water stress. ARAC3 encodes a Rho-like GTP binding protein which regulates resistance and microtubule ordering in *Arabidopsis* during powdery mildew pathogen attack [80]. In the present study induced expression of ARAC3 in resistant plants following Foc1 attack probably indicates the host’s attempt to modulate the Rho mediated signaling for protecting structural cohesiveness of the invaded cells. CSN5A encodes a subunit of the COP9 complex that are known to regulate neddylation-deneddylation pathways that modulate development as well as defense [81]. Downregulation of CSN5A homologue in resistant plants following Foc1 attack emphasizes on the need for degradation of targeted proteins that presumably reinforce resistance. GRF2 a class of 14-3-3 proteins acts as phosphosensors to regulate plant pathogen interactions by controlling pathogen perception, targeting defense proteins and/or pathogen effectors all of which are case specific [82]. Interestingly, in the present study, GRF2 were found to be downregulated in resistant plants following infection with Foc1. Such suppression of GRF2 was in all probability associated with enhanced resistance in chickpea during Foc1 attack, although the mode of action of GRF2 remains unclear. RAP2.3 (Ethylene-responsive element binding protein, related to AP2.3) is known to regulate ethylene and ABA response synergistically during low oxygen, osmotic stress and oxidative stressful conditions in *Arabidopsis thaliana* [83]. In present study, RAP2.3, was found to be upregulated in resistant variety following Foc1 infection suggesting its role in protecting the host from the devastating effects of osmotic and oxidative stress (Table 1, Fig 10, S8 Table).

Apart from the above components, several heat shock proteins and heat shock factors were found to be upregulated in chickpea both during compatible as well as incompatible interactions with Foc1. Heat shock proteins and factors are essential signaling molecules modulating intracellular signaling during diverse stressful situations. Although the relation of thermotolerance and plant immunity is unclear, but heat tolerance is believed to have connections with oxidative stress occurring as an invariable phenomenon linked to pathogen invasion [84]. HSF1 (Heat shock factor 1) is believed to regulate the expression of the defensin gene *Pdf1*.*2a/b* in *Arabidopsis thaliana* during infection caused by necrotrophic pathogen *Alternaria brassicicola* [85]. The expression of HSF1 in both susceptible and resistant plants following Foc1 infection suggests its common regulatory mechanism to prevail in both systems which is probably linked more to pathogen attack than to regulating defense response. HSP70 and HSP101 (Heat shock protein 70 and 101) act as molecular chaperones regulating protein folding and provide elevated tolerance against drought and saline stressed situations [84]. Moreover, HSP70 controls unfolded protein response (UPR) which is caused by accumulation of misfolded proteins during biotic and abiotic stress [86]. In present study, upregulation of HSP70 and 101 in susceptible plants following Foc1 predicts the role of HSP signaling in compatible plants to be somewhat under pathogenic controls, while HSF1 has generalized function in both the compatible and incompatible systems (Table 1, Fig 10, S8 Table).

### Protein synthesis/degradation and structural genes are altered under pathogen attack

Protein turnover of host impacts on the outcome of defense against biotic agents [87]. In the present study several transcripts related to protein synthesis and degradation were identified following infection with Foc1.

FKBP15-2 encodes for a peptidyl prolyl cis trans isomerase that are known to catalyze the isomerization reaction and help in folding of newly synthesized proteins. However, recent studies on rice showed the role of these compounds in targeting Aux/IAA for proteosomal degradation during auxin signaling [88]. Hence, such studies highlight the role of these isomerases in controlling not only protein folding but also protein degradation. In the present study, the upregulation FKBP15-2 in susceptible plants following Foc1 attack directs towards their role in protein degradation. But, how that adds to pathogen fitness is unclear. The ubiquitin-proteasome system is referred to as the central modifier in plant signaling [89]. Ubiquitin mediated degradation regulates detection of PAMPs by host PRRs, controls HR mediated cell death, regulates the degradation of transcription factors controlling calcium signaling etc [90]. Upregulation of UBQ1 (Ubiquitin 1) in susceptible plants and UBQ10 and UBQ35 in resistant plants post inoculation emphasizes on the universal role of ubiquitins in modulating protein turnover during pathogen encounter without having any specific role in deciding about the pathogenic outcome. On the contrary, RUB1 (Related to ubiquitin 1) which regulates protein degradation by complexing with SCF ubiquitin ligase complex was found to induce chitinase and defensin gene *PDF1*.*2* in *Arabidopsis thaliana*. Thus, increment of RUB1 in susceptible plants predicts the induction of chitinase which was reported to act as negative regulator of defense in chickpea-Foc1 case study [17] (Table 1, Fig 11, S8 Table).

PaB1 (encodes 20S proteasome subunit) involved in ubiquitin dependent catabolic process was reported to be induced during infection caused by *Pseudomonas syringae* in *Arabidopsis thaliana* [91]. However, the decrement of PaB1 in resistant plants following Foc1 attack directs towards its somewhat different role in present case study. RPT2A (encodes for 26S proteasome AAA-ATPase) was found to directly interact with CC-NBS-LRR type R protein in *Arabidopsis thaliana* [92]. Interestingly, in present study, the upregulation of RPT2A in resistant plants focuses on the importance of the study to look into the binding partner of RPT2A that could lead to the identification of R protein which is still unidentified from chickpea-Foc1 case study. PBE1 (component of 20S proteasome subunit) was reported to be induced and impart resistance against *Phytopthora cinnamomi* by regulating auxin signaling in *Arabidopsis thaliana* [93]. In present study, downregulation of PBE1 in resistant plants suggest a somewhat different function of PBE1, where resistance is imparted probably by shielding the targeted degradation of some important proteins. HDA19 (Histone deacetylase 19) have important role in controlling chromatin structure and gene expression, which in turn contribute to regulated protein production. Previous studies suggest negative role of HD19 in regulating SA mediated expression of PR1 and PR5 during biotrophic interaction [94]. Additionally histone deacetylase also negatively regulates plant immunity by decreasing H4 acetylation that leads to enhanced susceptibility against *Magnaporthe oryzae* and *Xanthomonas oryzae* p.v *oryzae* in rice [95]. Down regulation of HD19 in susceptible plants predicts on its negative regulatory role that assists to Foc1 establishment. T6D22.3 (Elongation factor EF1A-3) like elongation factors are abundantly found in cells. They possess unique functional dichotomy in regulating both protein synthesis as well as degradation. They recognize damaged protein and shuttle them for proteasome degradation. Besides they are known to regulate protein folding and are prominent regulators providing resistance to apoptosis following growth factor withdrawal [96]. Increase in amount of EF1A-3 in resistant plants suggest their role in assisting host defense by restricting apoptosis at the central vasculature of the host (Table 1, Fig 11, S8 Table).

EMB2780 DNA polymerases are well known for their roles in DNA repair which ultimately affect the translation process and protein quantum. Studies show that homologous recombination based repair mechanism are enhanced in plants exposed to biotic stresses and often the changes incurred are conserved as epigenetic modifications for ensuring better adaptation over the generations [97]. Upregulation of DNA polymerase in both susceptible and resistant plants showed common DNA damage responses under Foc1 attack.. LBA1 (Regulator of nonsense transcript like protein) proteins initiate nonsense mediated mRNA decay (NMD) which is conserved mechanism that target aberrant mRNAs for decay. Pathogen responsive genes including SA responsive molecular markers genes and PR genes were upregulated in NMD deficient plants in *Arabidopsis thaliana* which showed partial resistance to *Pseudomonas syringae* [98]. Enhanced expression of LBA1 in susceptible plants in the present study explains their probable role in regulating NMD mediated suppression of resistance responses (Table 1, Fig 11, S8 Table).

Many structural proteins are also altered during pathogen infection. LHB1B1 and LHCA2 (Chlorophyll a/b bonding proteins) are abundant class of proteins that are affected by light, oxidative stress, chlorophyll retrograde signaling, circadian clock and ABA. Reports on *Arabidopsis thaliana* suggest that the downregulation of these proteins result in accumulation of ROS which is decreased by ABA under drought stressed condition [99]. In present study down regulation of both LHB1B1 and LHCA2 in resistant plants suggest an attempt to minimize energy utilization by lowering photosynthetic demands of roots. On the other hand such lowering of chlorophyll binding proteins could also have a role in ROS detoxification and/or stomatal movement thus preventing water loss due to Foc1 induced wilt. ELIP1 (Early light inducible protein 1) serve as protectants during photooxidative stress [100]. However, the role of ELIP1 in defense of root tissues is unclear (Table 1, Fig 11, S8 Table).

GCP2 (Gamma tubulin protein complex 2) contributes in microtubule nucleation that leads to the formation of organized microtubule arrays of cellular cytoskeleton [101]. Pathogenic ingress causes an obvious alteration in structure and organization of microtubule assembly. Besides, previous reports also suggested that microtubule depolymerization leads to inhibition of hypersensitive response and imparts resistance to flax plants during infection caused by *Melamspora lini* [102]. Similarly, in present study, the decrement of GCP2 in resistant plants indicates towards microtubular depolymerization that probably blocks HR at the vascular site of infection. In contrast, another important regulator of cytoskeletal organization TUA6 (Tubulin alpha 6) was found to be upregulated in susceptible plants. Tubulin alpha chain protein was found to be upregulated in *Vitis vinifera* during infection by biotrophic oomycete *Plasmopara viticola* [103]. However, enhanced expression of TAU6 in susceptible plants suggests Foc1 governed alteration in cytoskeletal organization which probably facilitated fungal spread. FLA12 (Fasciclin like arabinogalactan protein 12) is arabinogalactan containing glycoprotein that modulates cell wall adhesion, cell to cell communication and formation of secondary cell walls [104]. Mutation of FLA proteins results in reduced tensile strength and cell wall elasticity leading to altered architecture and composition [105]. Decrement of FLA12 in susceptible plants indicates damages of cell wall integrity which was presumably a resultant of Foc1 establishment (Table 1, Fig 11, S8 Table).

IRX3 (Irregular xylem 3) belongs to cellulose synthase group of proteins that regulate synthesis of cellulose microfibrils that make up for primary cell walls. Downregulation of this class of protein leads to disruption of cell wall which makes the host susceptible to pathogen attack [106]. Hence, downregulation of IRX3 in susceptible plants predicts damage of cell wall as a consequence of Foc1 establishment. ATFH8 (Formin 8) encodes for a formin like protein that helps in nucleation of actin proteins and also regulates intracellular signaling [107]. However, in the present study, down regulation of this protein in resistant plants focuses on its role in probably controlling intra cellular signaling which needs further investigation. NifU4 regulates iron sulfur cluster assembly of mitochondria suggesting an indirect role in energy production [108]. Upregulation of NifU highlights constant need for energy generation to counteract pathogen destruction. SMC2 (Structural maintenance of chromosome 2) is known to be involved in chromosome condensation, regulation of higher order chromatin structure apart from coordinating DNA repair [109]. In the present study upregulation of SMC2 in resistant plants suggest an attempt to protect self from pathogenic devastations by deploying repair mechanisms (Table 1, Fig 11, S8 Table).

### Transport related genes are important players coordinating defense network

Transportation of essential elements at primary locations at needful hour controls the resistance network where diverse group of transporters play vital roles. NRT1.1 (Nitrate transporter) and NIA2 (Nitrate reductase) are known to control nitric oxide (NO) biosynthesis and transport. However, the role of NO in defense is quite mysterious as it has been found to favour both the invader and the host and at times reported to have dual roles which depends who takes command over whom and when during the course of pathogenetic events [110]. NRT1.1 and NIA2 both are reported to control stomatal opening and closure during drought stress which adds to susceptible changes in *Arabidopsis* [39]. In the present study down regulation of both NRT1.1 and NIA2 in resistant plants supports the fact that it was a probable attempt of the resistant host to deflect drought stress that was caused by the phenolic depositions induced by Foc1 invasion. ATGCN (*Arabidopsis* ABC transporter belonging to GCN superfamily) was also reported control stomatal and/or apoplastic defenses against *Pseudomonas syringae* p.v tomato DC3000 in *Arabidopsis* [111]. In present study the vascular wilting caused by Foc1 suggests some overlapping alterations to take place as during drought stressed conditions where the stomatal performance and changes are accountable. CHX20 (K+/proton antiporter) participates in K+ homeostasis through fluxes at intracellular compartments. Besides they are also known to regulate stomatal opening apart from facilitating membrane dynamics, vesicle budding trafficking needed to maintain turgor pressure at guard cells [112]. Upregulation of CHX20 in resistant plants emphasizes its regulatory role in imparting defense against Foc1 probably by counter balancing the flow of K^+^/proton (Table 1, Fig 12, S8 Table).

SKD (Suppressor of K+ transport growth defect 1) represent an ATP consuming vacuolar sorting protein 4 (VSP4). It is the core component of ESCRT pathway (Endosomal sorting complex for transport) that degrades plasma membrane proteins. Ubiquitination of appropriate plasma membrane protein cargos are important in regulating development and stress [113]. Besides, upregulation of SKD is linked to expression of PR1in *Arabidopsis* [114]. In the present study increased accumulation of SKD in resistant plants after infection suggests ubiquitination mediated degradation of specific plasma membrane protein that probably hinder the resistance mechanism. SUC2 (Sucrose proton symporter 2) are reported to serve as sucrose transporters facilitating the phloem loading/unloading in roots. Reports of *Arabidopsis thaliana* suggest that during water deficit conditions SUC2 are upregulated for allocating relatively more carbon to the roots for maintaining their normal metabolic activities [115]. Decrement of SUC2 in susceptible plants highlights their failure to promote carbon influx in roots during Foc1 induced water stressed condition that probably aided the plant to surrender to fungal ingress. On the contrary, resistant plant had better management strategies that made their basal metabolism function to the optimum. Both Gamma TIP (Tonoplast intrinsic protein) and Delta TIP aquaporin proteins are known to regulate water stress by facilitating water transport. In addition reports suggest that their down regulation leads to senescence and cell death [116]. In the present study, downregulation of both transporters in compatible plants probable indicate the onset of senescence which led to gradual susceptibility (Table 1, Fig 12, S8 Table).

## Conclusion

**Fig 13:**
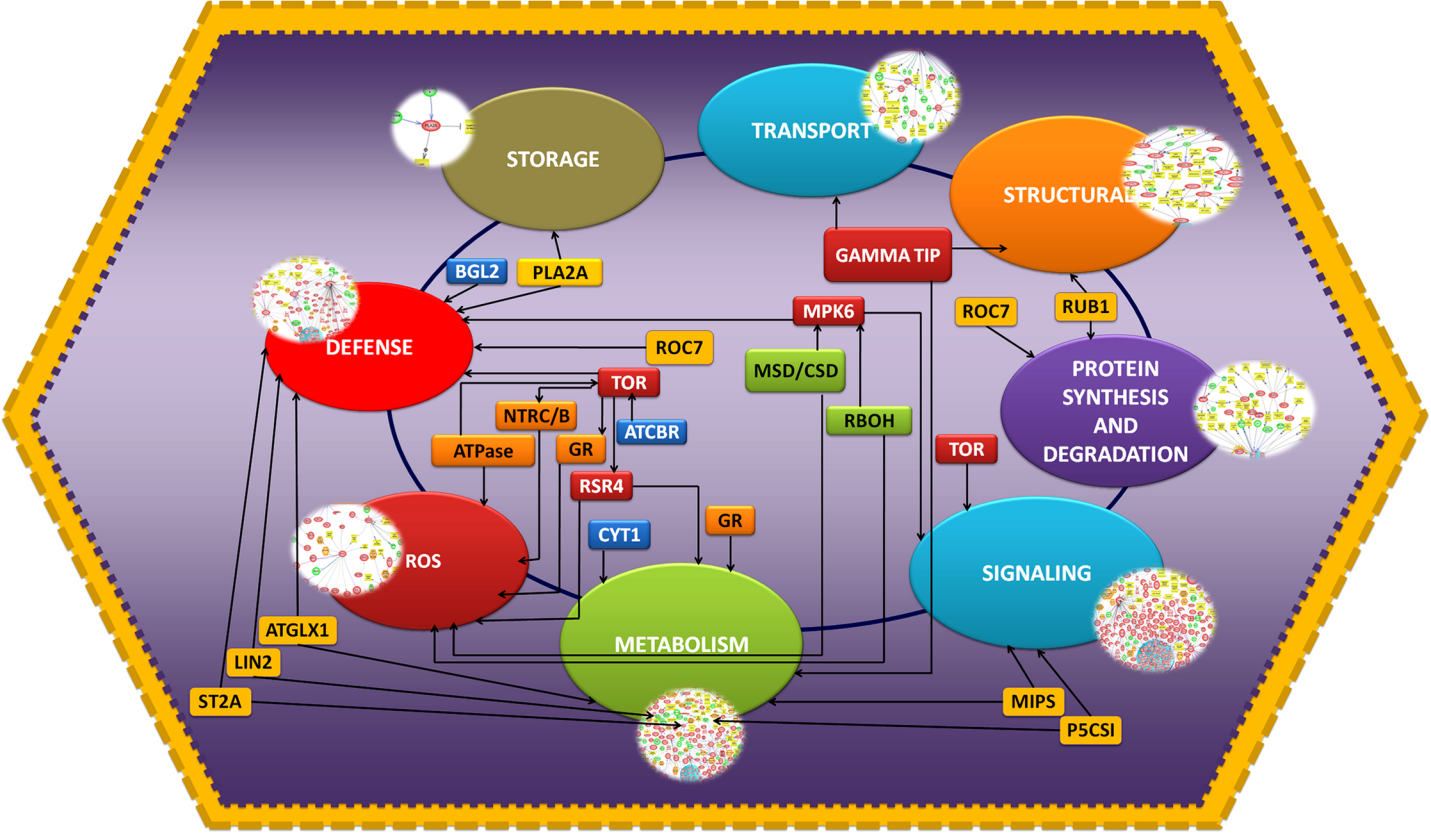
Composite integrated network showing the different nodal molecular components. Nodal key components belonging to different biological classes such as defense, ROS, metabolism, signaling, protein synthesis and degradation, transport and storage that have overlapping roles in chickpea defense against Foc1 attack. Rectangular boxes represent nodal molecules.

The present study highlights the regulatory complexities of defense responses in chickpea during Foc1 infection. The study identifies several differentially expressed components regulating the host-pathogen interaction network. However, amongst them, handful components were found to share overlapping roles. For example ROC7 regulated folding and synthesis of defense related proteins. MPK6 regulated defense response, signaling as well as ROS metabolism by interacting with RBOH, MSD and CSD. BGL2 had role in regulating defense and modulating metabolism by influencing the expression of CYT1. TOR was found to be a direct regulator of defense and ROS through its connection with ATCBR, RSR4, GR, NTRC/B and ATPase. ATGLX1, LIN2 and ST2A had overlapping role in defense and metabolism while PLA2A being a storage component also regulated defense. Gamma-TIP, a structural component regulated both metabolism and transport. MIPS and P5CS1 had roles controlling signaling as well as metabolism. RUB, a structural component regulated protein synthesis and degradation (Fig 13).

Therefore, in the entire Chickpea-Foc1 interaction network, the above mentioned molecules with overlapping roles probably modulated the defense signaling network by forming signal controlling nodal hubs. These hub components with defined mode of action may pose themselves as promising candidates for resistant management programs of legumes using genetic engineering technique.

## Materials and methods

### Plant material and fungal culture

Wilt susceptible (JG62) and wilt resistant (WR315) chickpea (*Cicer arietinum* L.) seeds obtained from International Crops Research Institute for Semi-Arid Tropics (ICRISAT), Patancheru, Andhra Pradesh, India were used for performing the experiments. Seeds were sown in mixture of sand: synthetic soil (1:1) and plantlets maintained under natural greenhouse conditions with temperatures ranging from 22–28°C, humidity 35–40%, and photoperiod of 16:8 hours day and light, respectively.

Fungal strain of *Fusarium oxysporum* f. sp. ciceri (Foc1), obtained from ICRISAT, purified and maintained according to Summerell, Salley & Leslie [117]. Spores were harvested and stored at -80°C in 30% glycerol for further use.

### Induced infection and selection of timepoint

Seeds of both JG62 and WR315 were surface sterilized and germinated in sterile synthetic soil. Fourteen days old seedlings were used for the infection assay. Infection with Foc1 was induced by sick soil method as described by Gupta et al [14]. Optimal growth conditions were provided to both inoculum free control and sick soil treated experimental sets. Previous experiments showed significant transcriptomic and proteomic alterations at 48 h post inoculation [15, 20]. Thus, 48 h was chosen as the optimal time point for sample collection (Fig 1). The collected root samples were weighed into 1 g aliquots, flash frozen in liquid nitrogen, and stored at -80°C for RNA extraction.

### RNA quality analyses and library construction

Library was constructed according to the Illumina TruSeq RNA library protocol outlined in “TruSeq RNA Sample Preparation Guide” (Part # 15008136; Rev. A; Nov 2010). RNA purity was analysed by calculating RNA integrating number (RIN) for both uninfected and infected samples prior to mRNA preparation (S6 Fig). RNA samples were extracted from three independent biological replicates and then pooled and subjected to downstream preparation. 1ug of total RNA was subjected to Poly A purification of mRNA. Purified mRNA was fragmented for 4 minutes at elevated temperature (94^°^C) in the presence of divalent cations and reverse transcribed with Superscript III Reverse transcriptase by priming with Random Hexamers. Second strand cDNA was synthesized in the presence of DNA Polymerase I and RnaseH. The cDNA was cleaned up using Agencourt Ampure XP SPRI beads (Beckman Coulter). Illumina Adapters were ligated to the cDNA molecules after end repair and addition of A base. SPRI cleanup was performed after ligation. The library was amplified using 11 cycles of PCR for enrichment of adapter ligated fragments. The prepared library was quantified using Nanodrop and validated for quality by running an aliquot on High Sensitivity Bioanalyzer Chip (Agilent).

### RNA-seq assembly and quality control

Illumina Hiseq 1000 platform was used to generate paired end short reads using Sequencing By Synthesis (SBS) method. Standard illumine pipeline (RTA-CASSAVA-OLB) was used to generate short reads in FASTQ format. Accuracy of base calling was reflected in the quality scores which were performed after filtering. Quality control was done using in-house program (Seqc V2.1- http://genotypic.co.in/SeqQC.html) to generate high quality reads for use in assembly. The reads were filtered or trimmed for adapters, B trimming and other low quality read trimming by standard methodology (CASAVA 1.7User Guide). These high quality, filtered reads were used for further analyses.

### Transcriptome assembly and clustering

Contig assembly for all the uninduced and induced samples of JG62 and WR315 was carried out using a de Bruijn graph algorithm based de novo genome assembler Velvet-1.1.07 (http://www.ebi.ac.uk/zerbino/velvet/). Draft assembly was built with fixed hash length for each sample. The values of the estimated insert length, insert length standard deviation and expected coverage for the all the draft assemblies were also calculated. The resulting contigs were assembled into transcripts by Oases-2.01 (http://www.ebi.ac.uk/zerbino/oases/), using the assembly of velvet that clustered them into small groups (loci). Paired end information was used to construct transcript isoforms. Assembly statistics were calculated using in-house Perl scripts. The transcripts from all assemblies were clustered (CD_HIT v 4.5.4 http://www.bioinformatics.org/cd-hit/) to generate a comprehensive de novo reference assembly. Sequence identity threshold and alignment coverage (for the shorter sequence) were both set to 80% to generate clusters. Such clustered transcripts are defined as reference transcripts in the present study.

### Functional annotation

Functional annotations of transcripts were carried out using Viridiplantae (Papilionoideae) database of mRNA datasets of NCBI. Although recent researches have identified the whole draft genome sequence of *Cicer arietinum* [6,7], but the annotations are still on track. Thus multiple databases were used to annotate the functional proteins arising from the obtained transcripts. Initial megablast searches were performed using Viridiplantae (Papilionoideae) database of mRNA datasets of NCBI database using E- value cut off of e-5. Further blastx were carried out against Swiss Prot, TrEMBL, KOG and PlantCyc enzymes. Blast annotations were filtered using subject to query coverage of 30% and sequence identity of 50% in case of megablast and 30% for blastx searches suites. InterProScan-4.8 (http://www.ebi.ac.uk/Tools/pfa/iprscan/) was used to scan Pfam database for identifying protein domains. Final annotation table was prepared giving preference to Swiss-Prot, PlantCyc and KOG databases following which GenBank Viridiplantae mRNA or TreEMBL annotations were assigned. Pfam annotations were assigned to only those trasnscripts that did not show matches with any of the above mentioned databases.

### Quantifying differential transcripts

Differential expression analyses were performed by employing a negative binomial distribution model (DESeq v 1.8.1 package http://www-huber.embl.de/users/anders/DESeq/). P value cutoff of 0.05 was used to filter statistically significant results. Fold change was calculated from the base mean value of treated samples with their corresponding control sample. P values were further adjusted maintaining standard parameters assigning specific Q values to P significant differential transcripts. Only Q significant transcripts were considered for downstream functional clustering and network generation.

### Functional clustering of transcripts and radar plot analyses

Following functional annotation using different database searches and alignments, the obtained functionally classified transcripts were subjected to clustering based on gene ontology. Besides they were also subjected to different sub division clusters such as ones related to direct defense, reactive oxygen species generation (ROS), metabolism, signaling, protein synthesis and degradation, transport, structural and storage.

The functional categorization of Q significant annotated transcript was performed using ChartTool software package. The number of transcripts in each category was used as an input to generate pie chart of total transcripts. Pyramiding of each category was also performed using individual transcript number of each group. The area of each stage of the pyramid corresponds to the abundance of transcript group of the category.

The expression patterns of the transcripts from the base mean value were demonstrated through radar plot analyses using Excel Stat software plug in. The fold change value of susceptible JG62 plants and resistant WR315 plants were obtained from base mean values of each transcripts after infection and its cognate control values. The transcripts that are expressed only after infection in both the susceptible and resistant plants without any cognate control values, the fold change was calculated by normalizing the data with base mean values of susceptible JG62 plants. A surface plot using all the fold change values of the entire transcript set was also performed.

### Quantitative real-time PCR (qRT-PCR)

Twenty transcripts from the NGS dataset were randomly selected and quantitative real time PCR (qRT-PCR) was carried out on Bio-Rad iCycler (Bio Rad iQ5) using SyBr green technology. Total reaction volume of 20 μl comprised of SyBr green qPCR Supermix (2X) (BioRad), 25ng cDNA and 0.3 μM sequence specific forward and reverse primers (S10 Table). PCR cycling conditions were as follows, a single cycle of pre heating at 95°C for 5 min, followed by 40 cycles at 95°C for 30 s, 50–55°C for 45 s and 72°C for 30 s [118]. Melt curve was analyzed to determine primer specificity and entire data set was normalized using GAPDH as internal control [118]. The mean fold change was calculated using Livak’s 2^-ΔΔ*C*^_T_ method [119].All experiments were conducted in triplicates and standard error was calculated for each transcript.

### Network analyses

Interaction network was generated using Pathway studio version 10.1 in two different ways. Initial network was generated by importing the entire experiment set bearing differential transcripts with significant P values (<0.05). Further sub networks were generated according to different sub divisional functional classes such as direct defense, reactive oxygen species generation (ROS), metabolism, signaling, protein synthesis and degradation, transport, structural and storage. In both cases differential transcripts identified by RNA-seq studies were subjected to BLAST analyses at TAIR database (The Arabidopsis Information Resource) and their homologous genes (bearing TAIR gene IDs, S11 Table) used as inputs for network construction. Ambiguities and components without any interactive neighbors were eliminated from the import list. Interaction network was generated using the neighbor joining method with a degree of correlation as 1 (only the immediate neighbors both upstream and downstream having straight relationship to the protein/protein products were considered for analyses). Besides, standard filter parameters and relation types were selected for interaction map construction.

### Abbreviations

All Abbreviations used in this article are provided in S9 Table and S11 Table.

## Supporting information

S1 Fig Anatomical details of chickpea roots after 48h of Foc1 infection. Sectional views of control and infected roots of chickpea plants JG62 _control_ (A), (B) and WR315 _control_ (E), (F); JG62_infected_ (C), (D) and WR315 _infected_ (G), (H) at 48 hours post infection stained with Trypan blue and lactophenol. Bars represents 10μ respectively.
(TIF)

S2 Fig Graphical representation of reads statistics. The bars here represent the number of reads obtained for samples JC (uninduced susceptible), J4 (induced susceptible), WC (Uninducedresistant), W4 (induced resistant). Blue bar represents raw reads and red represents processed reads.
(TIF)

S3 Fig Graphical representation of contig statistics. The bars here represent the number of contigs of variable base pairs for J4 represented by blue bar, JC represented by red bar, W4 represented by green bar, WC represented by violet bar.
(TIF)

S4 Fig Graphical representation of transcript statistics. The bars here represent the number of transcripts of variable base pairs for J4 represented by blue bar, JC represented by red bar, W4 represented by green bar, WC represented by violet bar.
(TIF)

S5 Fig Pie chart representing the percentage distribution of differential transcripts. A. Percentage of transcript down regulated only in JG62 (blue), only in WR315 (red) and in both JG62 and WR315 (green). B. Percentage of transcript upregulated only in JG62 (blue), only in WR315 (red) and in both JG62 and WR315 (green).
(TIF)

S6 Fig Graphical representation of RIN (RNA integrity number) values. A. represents RNA integrity of JC (uninduced susceptible), B. represents RNA integrity of J4, C. represents RNA integrity of WC (uninducedreesistant), D. represents RNA integrity of W4 (induced resistant).
(TIF)

S1 Table Read statistics. Table containing read statistics of JC, J4, WC, and W4.
(XLS)

S2 Table Contig statistics. Table containing contig statistics of JC, J4, WC, and W4.
(XLS)

S3 Table Transcript statistics. Table containing transcript statistics of JC, J4, WC, and W4.
(XLS)

S4 Table Representative transcript statistics. Table containing representative transcript number and sizes between of JC and J4, WC and W4 respectively.
(XLS)

S5 Table Transcript annotation. Table containing transcript annotation statistics.
(XLSX)

S6 Table Q-significant values between JC and J4. (XLS)

S7 Table Q-significant values between WC and W4. (XLS)

S8 Table Gene list. Table includes gene list of down regulated and up regulated transcripts in chickpea upon foc1 infection.
(XLSX)

S9 Table List of abbreviations used in radar Plot. Table containing list of protein names and their abbreviation used in the Radar plot.
(XLSX)

S10 Table List of primers used for qRT-PCR analyses. Table includes list of primer pairs sequences used for qRT-PCR for representative transcripts with their respective abbreviations.
(XLSX)

S11 Table List of abbreviations used in the network generation. Table containing list of proteins, their abbreviations used for pathway construction and TAIR homologous IDs of the identified proteins used as input for network generation.
(DOC)

S1 File Protein network generation using pathway studio software version 7.1. The document shows interaction of proteins belonging to different biological classes such as defense, metabolism, protein synthesis and degradation, reactive oxygen species generation, signaling and storage in chickpea obtained post infection with Foc1.
(PDF)
