# Supplementary figures and images for: Transcriptomic dissection reveals wide spread differential expression in chickpea during early time points of Fusarium oxysporum f. sp. ciceri Race 1 attack
Source: PLoS One. 2017 May 25;12(5):e0178164. doi: 10.1371/journal.pone.0178164 (PMC5460890; doi:10.1371/journal.pone.0178164)

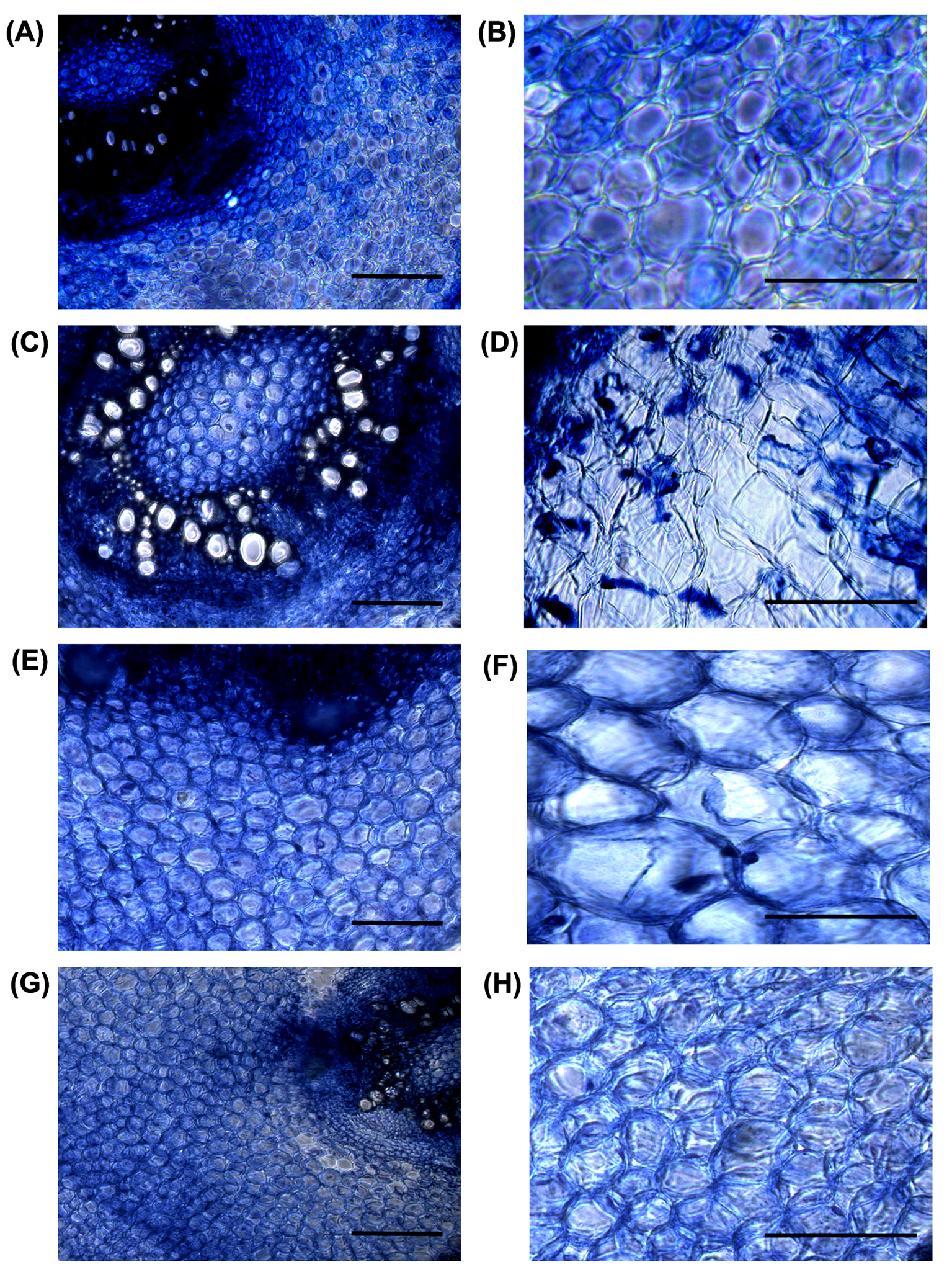

Supplement: S1 Fig — Sectional views of control and infected roots of chickpea plants JG62 control (A), (B) and WR315 control (E), (F); JG62infected (C), (D) and WR315 infected (G), (H) at 48 hours post infection stained with Trypan blue and lactophenol. Bars represents 10μ respectively. (TIF) [file pone.0178164.s001.tif]

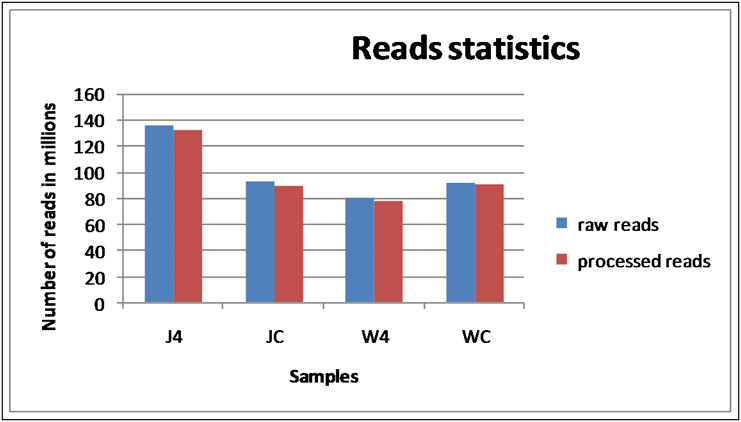

Supplement: S2 Fig — The bars here represent the number of reads obtained for samples JC (uninduced susceptible), J4 (induced susceptible), WC (Uninducedresistant), W4 (induced resistant). Blue bar represents raw reads and red represents processed reads. (TIF) [file pone.0178164.s002.tif]

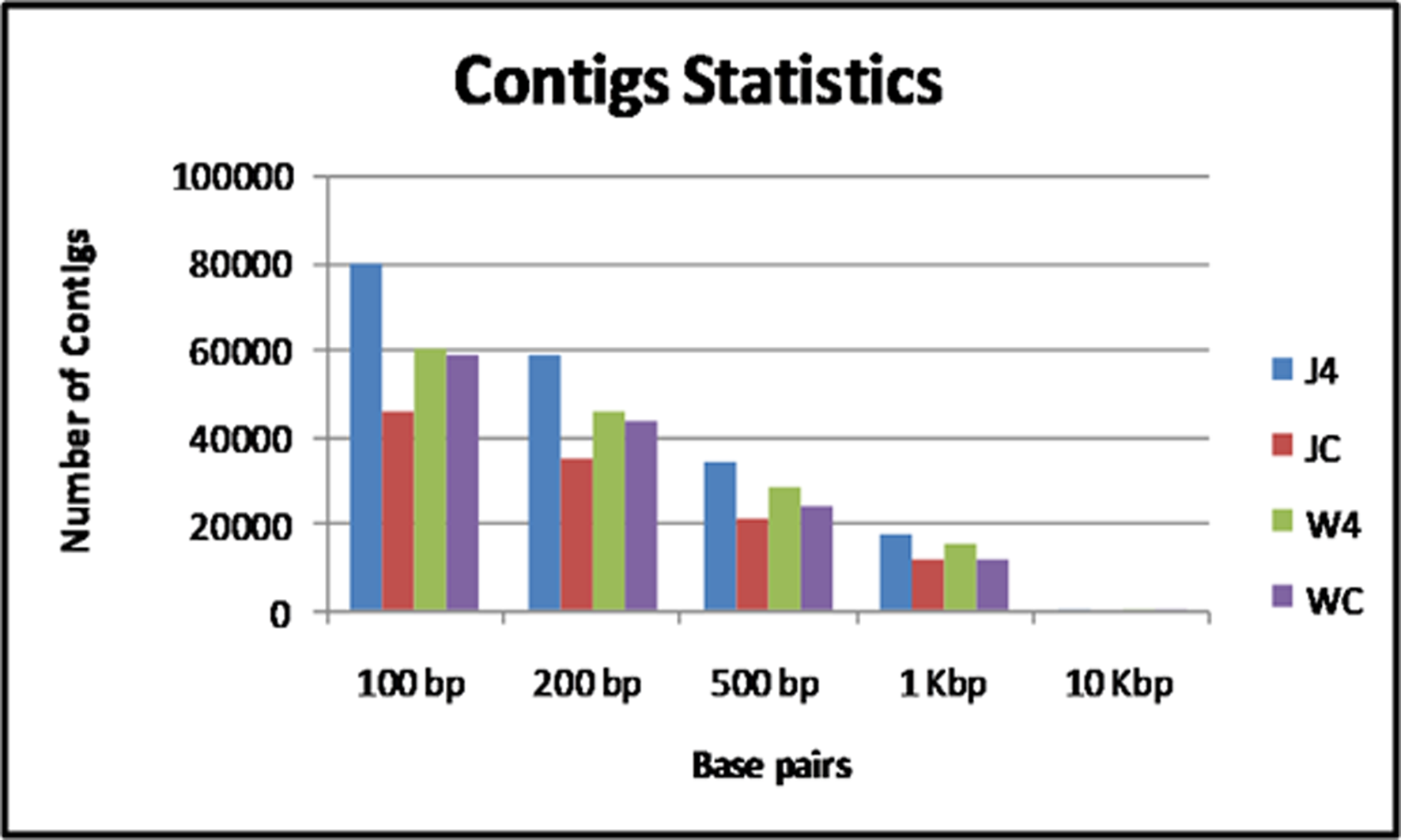

Supplement: S3 Fig — The bars here represent the number of contigs of variable base pairs for J4 represented by blue bar, JC represented by red bar, W4 represented by green bar, WC represented by violet bar. (TIF) [file pone.0178164.s003.tif]

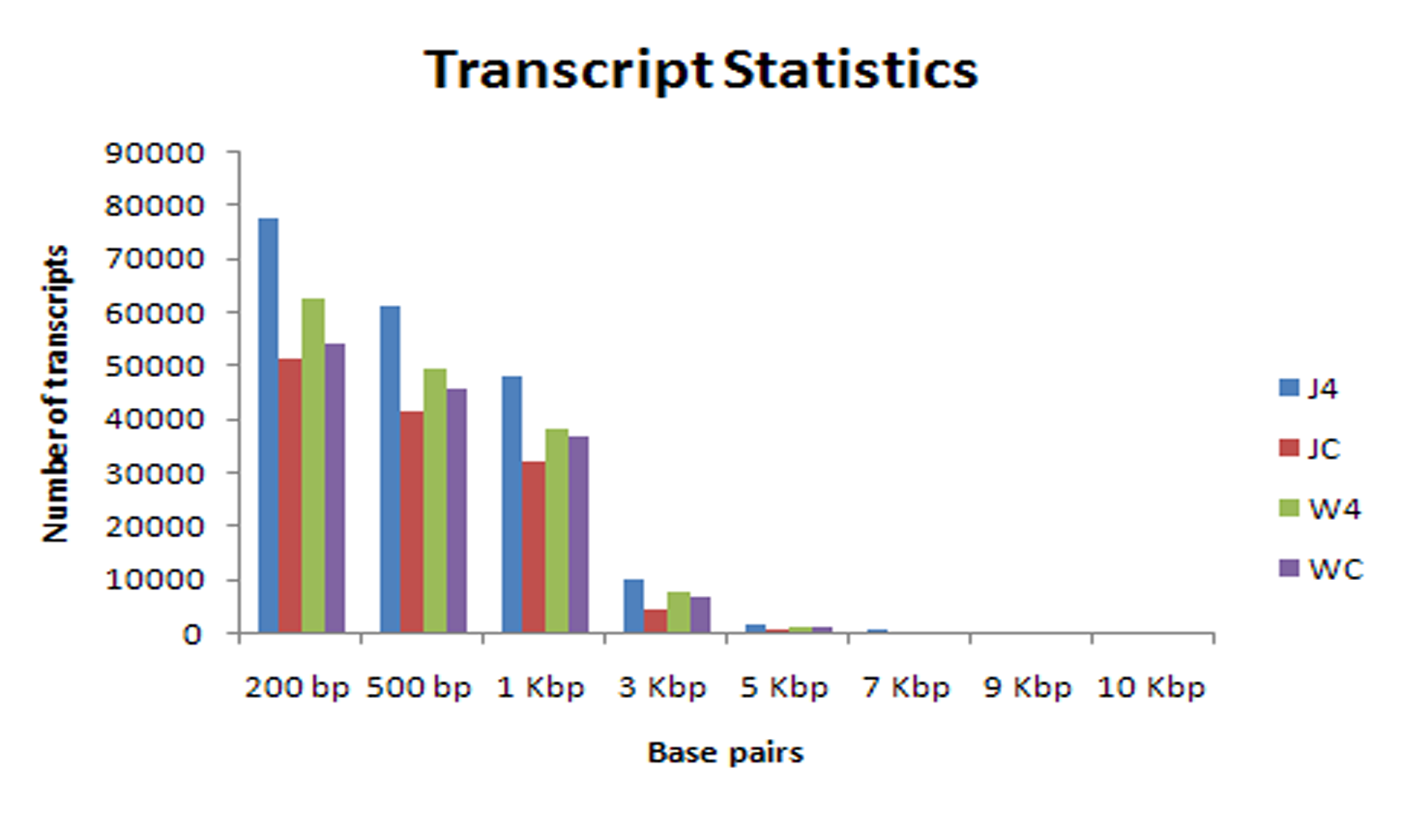

Supplement: S4 Fig — The bars here represent the number of transcripts of variable base pairs for J4 represented by blue bar, JC represented by red bar, W4 represented by green bar, WC represented by violet bar. (TIF) [file pone.0178164.s004.tif]

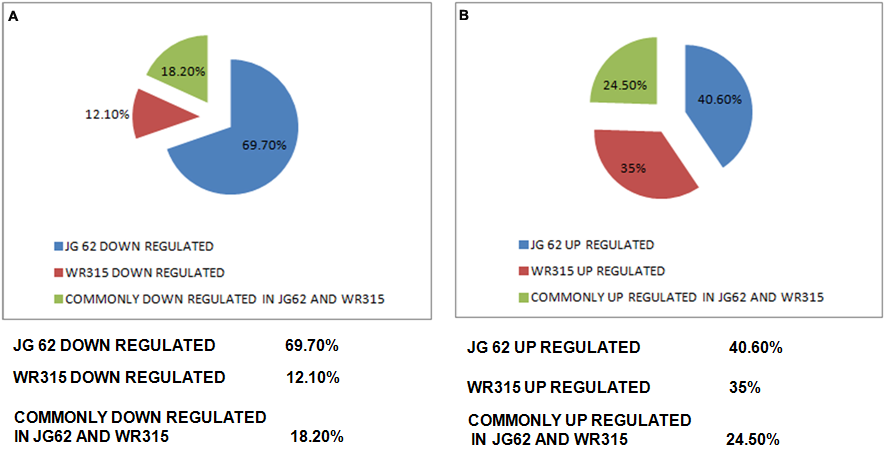

Supplement: S5 Fig — A. Percentage of transcript down regulated only in JG62 (blue), only in WR315 (red) and in both JG62 and WR315 (green). B. Percentage of transcript upregulated only in JG62 (blue), only in WR315 (red) and in both JG62 and WR315 (green). (TIF) [file pone.0178164.s005.tif]

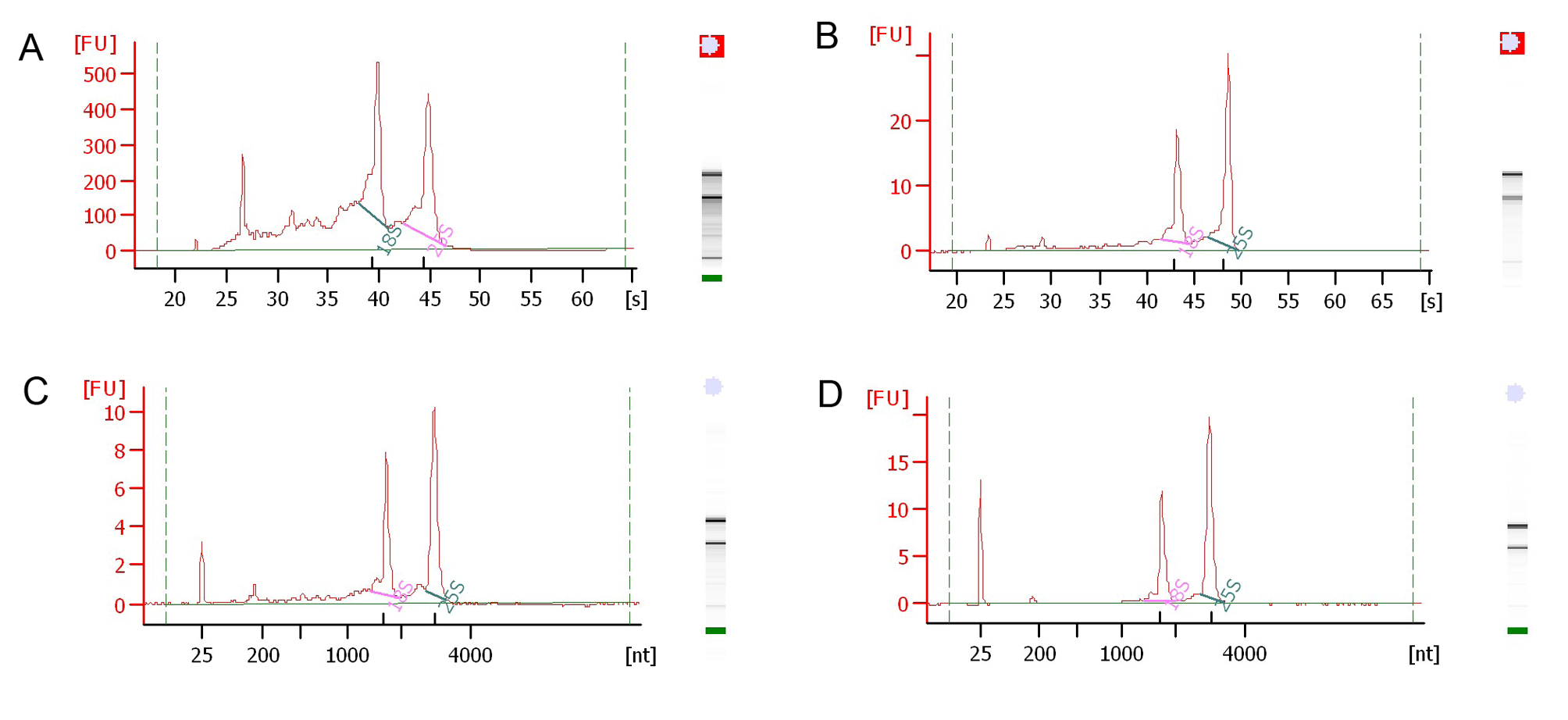

Supplement: S6 Fig — A. represents RNA integrity of JC (uninduced susceptible), B. represents RNA integrity of J4, C. represents RNA integrity of WC (uninducedreesistant), D. represents RNA integrity of W4 (induced resistant). (TIF) [file pone.0178164.s006.tif]
