# Supplementary material for: Transcriptomic dissection reveals wide spread differential expression in chickpea during early time points of Fusarium oxysporum f. sp. ciceri Race 1 attack
Source: PLoS One. 2017 May 25;12(5):e0178164. doi: 10.1371/journal.pone.0178164 (PMC5460890; doi:10.1371/journal.pone.0178164)
Supplement: S11 Table — Table containing list of proteins, their abbreviations used for pathway construction and TAIR homologous IDs of the identified proteins used as input for network generation. (DOC) [file pone.0178164.s017.doc]

**S11Table. List of abbreviations used in the pathway generation**

| **Protein abbreviation** | **TAIR ID** | **Protein name** |
| --- | --- | --- |
| **Defense** |  | |
| ROC7 | AT5G58710 | Peptidyl prolyl cis trans isomerase |
| BGL2 | AT4G26140 | Beta glucosidase |
| TOR | AT1G50030 | Serine threonine protein kinase |
| Oschib | AT3G12500 | Chitinase |
| ATGLX1 | AT1G11840 | Lactolylglutathione lyase |
| CSLD3 | AT3G03050 | Cellulose synthase |
| ST2A | AT5G07010 | Sulfotransferase |
| LIN2 | AT1G03475 | Coproporphirinogen III oxidase |
| MPK6 | AT2G43790 | MAP kinase |
| PGIP | AT5G06860 | Poly galactouronase inhibiting protein |
| T9A4.6 | AT4G10270 | Wound responsive protein |
| **ROS** |  | |
| Atp1 | ATMG01190 | V type ATPase |
| VHA-A | AT1G78900 | V type ATPase |
| ATCBR | AT5G17770 | Cytochrome b5 reductase |
| RSR4 | AT5G01410 | Reduced sugar response 4 |
| NTRC/B | AT2G41680 | NTRC/ NADPH-dependent thioredoxin reductase C/B |
| T518.3 | AT5G08680 | ATPase |
| MSD | AT3G10920 | Manganese Superoxide dismutase |
| CSD | AT5G18100 | Copper Superoxide dismutase |
| RBOH | AT1G09090 | Respiratory burst oxidase |
| **Storage** |  | |
| PLA2A | AT2G26560 | Patatin like protein |
| **Metabolism** |  | |
| MEE58 | AT4G13940 | S-adenosyl-L-homocysteine hydrolase |
| CYT1 | AT2G39770 | cytokinesis defective 1 |
| GLT1 | AT5G53460 | glucose transporter 1 |
| MTO3 | AT3G17390 | methionine over-accumulator 3, |
| APR3 | AT4G21990 | APS reductase 3 |
| C4H | AT2G30490 | cinnamate 4-hydroxylase |
| LOX1 | AT1G55020 | Lipoxygenase 1 |
| PCK1 | AT2G42600 | phosphoenolpyruvate carboxykinase |
| CWINV1 | AT3G13790 | cell wall invertase 1 |
| ADH1 | AT1G77120 | Alcohol dehydrogenase |
| ACO1 | AT2G19590 | acc oxidase 1 |
| MLS | AT5G03860 | malate synthase |
| ASN1 | AT3G47340 | glutamine-dependent asparagine synthase 1 |
| RNR1 | AT2G21790 | ribonucleotide reductase 1 |
| HOT5 | AT5G43940 | sensitive to hot temperatures 5 |
| SHM1 | AT5G26780 | serine hydroxymethyltransferase 1 |
| FDH | AT5G14780 | formate dehydrogenase |
| P5CS1 | AT2G39800 | delta1-pyrroline-5-carboxylate synthase 1 |
| LIP1 | AT2G20860 | lipase 1 |
| SDH1 | AT5G66760 | succinate dehydrogenase |
| RHM1 | AT1G78570 | rhamnose biosynthesis 1 |
| AMY1 | AT4G25000 | alpha-amylase-like |
| SBE2.2 | AT5G03650 | starch branching enzyme 2.2 |
| MTLPD2 | AT3G17240 | lipoamide dehydrogenase 2 |
| MIPS2 | AT2G22240 | myo-inositol-1-phosphate synthase 2 |
| IPP2 | AT3G02780 | isopentenyl pyrophosphate:dimethylallyl pyrophosphate isomerase 2 |
| GDH3 | AT3G03910 | glutamate dehydrogenase 3 |
| TIM | AT1G12900 | Glyceraldehyde dehydrogenase phosphate |
| IVD | AT3G45300 | isovaleryl-coa-dehydrogenase |
| PMDH1 | AT2G22780 | peroxisomal nad-malate dehydrogenase 1 |
| ADSS | AT3G57610 | adenylosuccinate synthase |
| GR | AT3G54660 | Glutathione reductase |
| SUS4 | AT3G43190 | Sucrose synthase 4 |
| **Signaling** |  | |
| CAM5 | AT2G27030 | Calmodulin protein 5 |
| CAM7 | AT3G43810 | Calmodulin protein 7 |
| HSF1 | AT3G12580 | Heat shock factor 1 |
| HSP101 | AT1G74310 | Heat shock protein 101 |
| HSP70 | AT3G12580 | Heat shock protein 70 |
| GRF2 | AT1G78300 | 14-3-3 G box binding protein |
| MYB5 | AT3G13540 | MYB transcription factor |
| MYB108 | AT3G06490 | MYB transcription factor |
| WRKY41 | AT4G11070 | WRKY transcription factor |
| RAP2.3 | AT3G16770 | Ethylene responsive transcription factor 2b |
| DREB1A | AT4G25480 | CRT/DRE binding factor 4 |
| NDPK2 | AT4G09320 | Nucleoside diphosphate kinase |
| CSN5A | AT1G22920 | COP9 Signalosome 5A |
| ARAC3 | AT4G35020 | GTPase |
| **Protein synthesis and degradation** |  | |
| FKBP15-2 | AT5G58710 | Peptidyl prolyl cis trans isomerase |
| UBQ1 | AT3G52590 | Ubiquitin 1 |
| UBQ10 | AT1G78870 | Ubiquitin 10 |
| UBQ35 | AT5G59300 | Ubiquitin 35 |
| PaB1 | AT1G16470 | Proteasome subunit |
| HD | AT4G38130 | Histone deacetylase |
| RPT2A | AT5G66140 | Proteasome component |
| T6D22.3 | AT5G60390 | Elongation factor |
| EMB2780 | AT5G63960 | DNA polymerase |
| LBA1 | AT5G47010 | Regulator of nonsense transcript like protein |
| RUB1 | AT3G05530 | Ubiquitin |
| PBE1 | AT1G13060 | Proteasome components |
| ATHMG | AT3G28730 | FACT comples subunit SSRP1 |
| **Structural** |  | |
| LHB1B1 | AT2G34430 | Chlorophyll a/b binding protein |
| LHCA2 | AT3G61470 | Chlorophyll a/b binding protein |
| Delta TIP | AT3G16240 | Delta Tonoplanst intrinsic protein |
| Gamma TIP | AT2G36830 | Gamma Tonoplanst intrinsic protein |
| ELIP1 | AT3G22840 | Early light inducible protein |
| GCP2 | AT5G05620 | Tubulin gamma chain |
| FLA12 | AT5G60490 | Fasciclin like arabinogalactan protein |
| IRX3 | AT5G17420 | Cellulose synthase |
| ATFH8 | AT1G70140 | Formin like protein |
| NFU4 | AT3G20970 | NifU like protein |
| TUA6 | AT4G14960 | Tubulin alpha chain |
| SMC2 | AT5G62410 | Structural maintenance of chromosome |
| F8L15.150 | AT5G08420 | KRR motif containing protein 1 |
| **Transport** |  | |
| CHX20 | AT3G53720 | K+/N+ antiporter |
| SKD | AT2G27600 | Vacuolar sorting protein |
| NRT | AT1G12110 | Nitrate transporter |
| ATGCN | AT1G64550 | ABC transporter family protein |
| SUC 2 | AT1G22710 | Sugar transporter |
