# Supplementary material for: Transcriptomic dissection reveals wide spread differential expression in chickpea during early time points of Fusarium oxysporum f. sp. ciceri Race 1 attack
Source: PLoS One. 2017 May 25;12(5):e0178164. doi: 10.1371/journal.pone.0178164 (PMC5460890; doi:10.1371/journal.pone.0178164)
Supplement: S1 File — The document shows interaction of proteins belonging to different biological classes such as defense, metabolism, protein synthesis and degradation, reactive oxygen species generation, signaling and storage in chickpea obtained post infection with Foc1. (PDF) [file pone.0178164.s018.pdf]

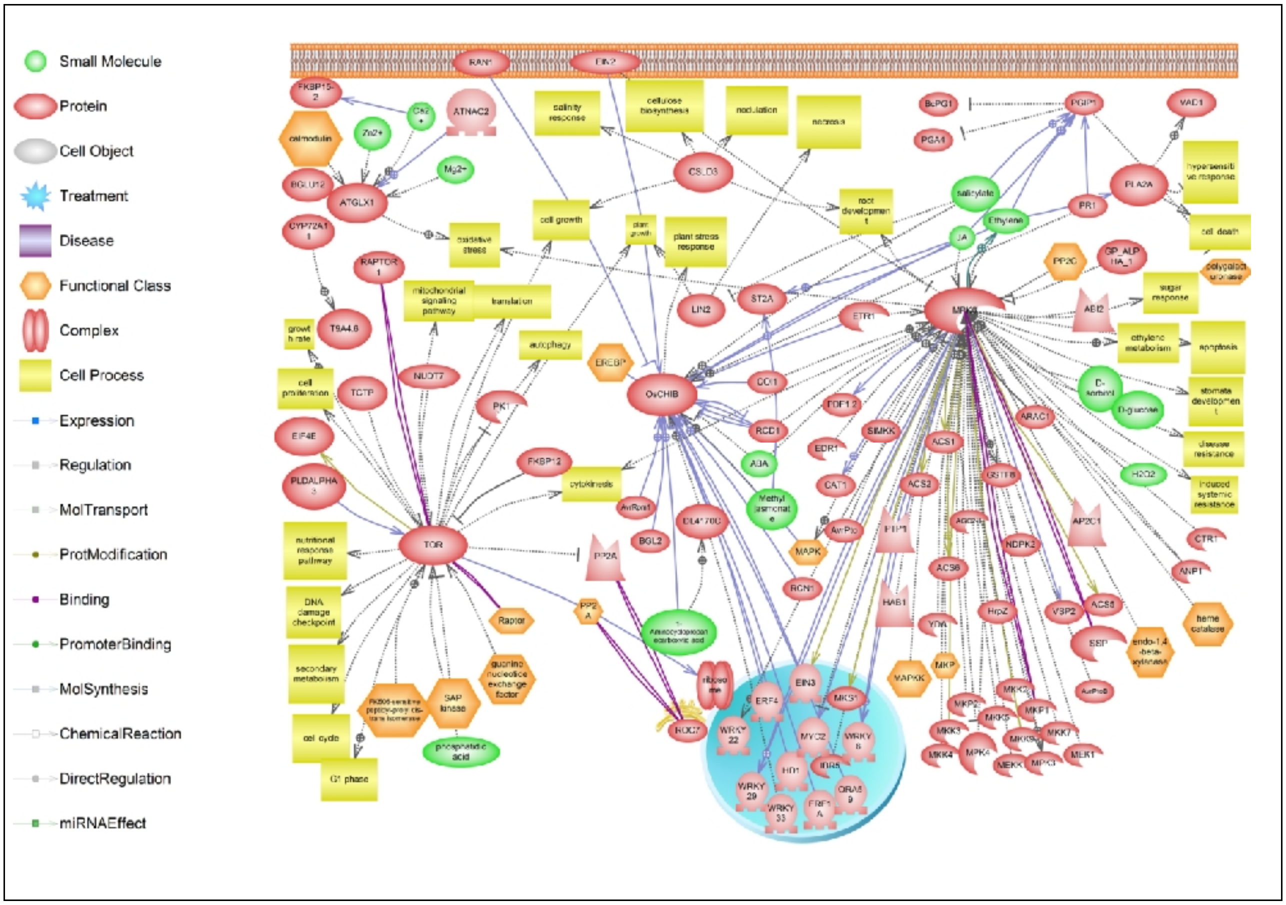

Figure 1.Network generation using pathway studio software version 7.1, network shows the interaction between defense related transcripts identified in chickpea after 48h post infection with Foc1.

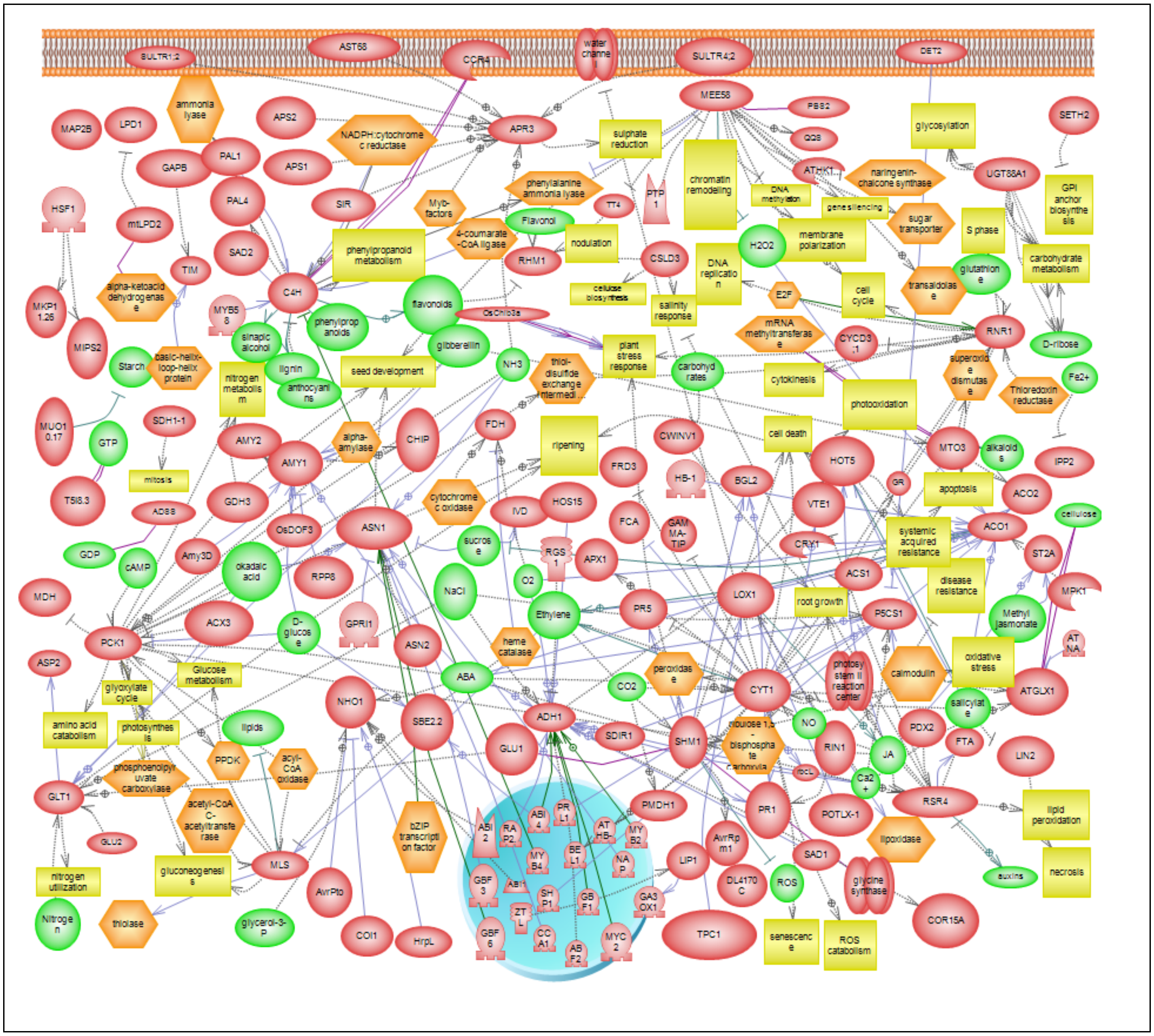

Figure2. **Network generation using pathway studio software version 7.1,** network shows the interaction between metabolism related transcripts identified in chickpea after 48h post infection with Foc1.

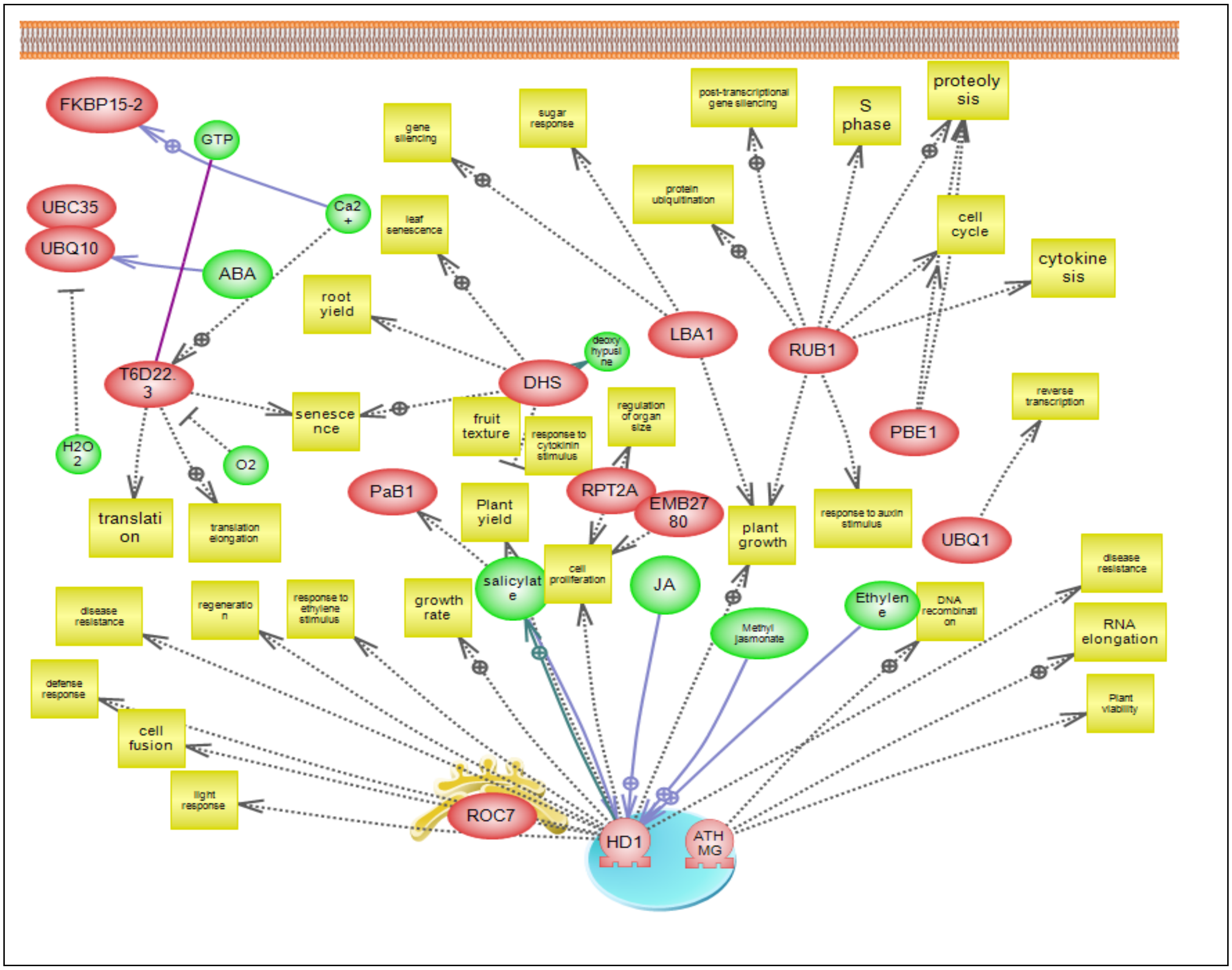

Figure3. **Network generation using pathway studio software version 7.1,** network shows the interaction between transcripts involved in protein synthesis and degradation, identified in chickpea after 48h post infection with Foc1.

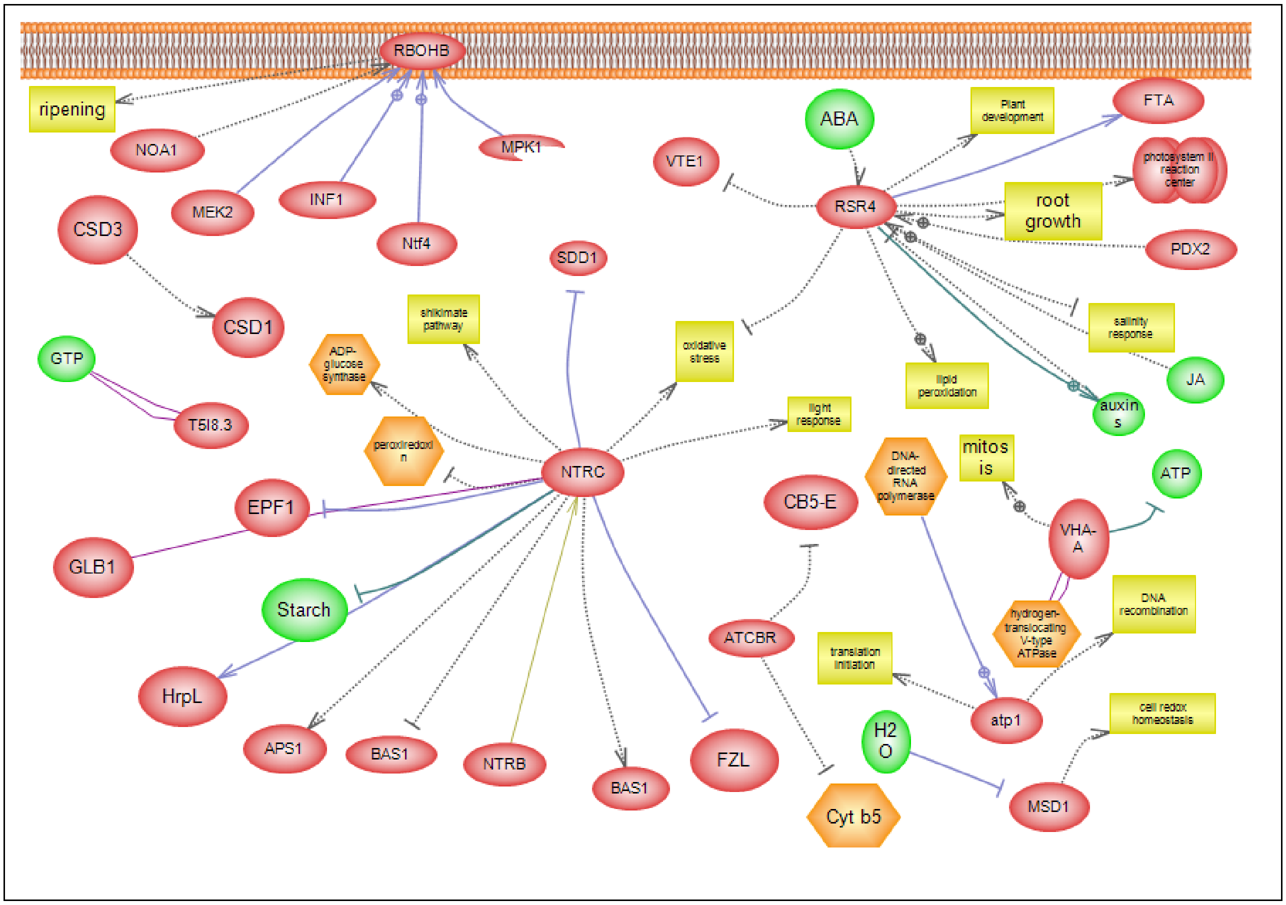

Figure4. Network generation using pathway studio software version 7.1, network shows the interaction between transcripts involved in Reactive oxygen species generation, identified in chickpea after 48h post infection with Foc1.

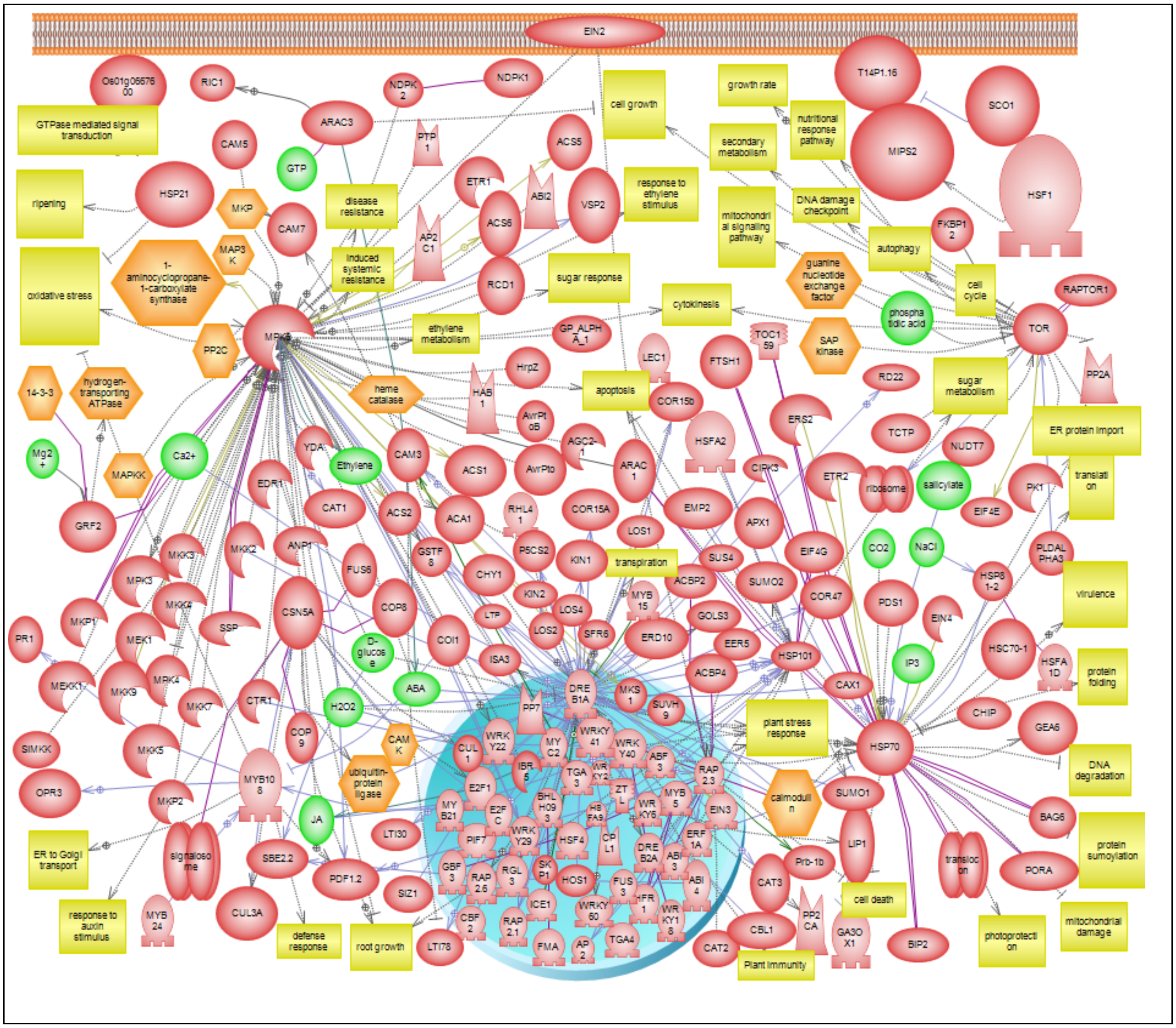

Figure5. Network generation using pathway studio software version 7.1, network shows the interaction between transcripts involved in signaling to regulate plant defense, identified in chickpea after 48h post infection with Foc1.

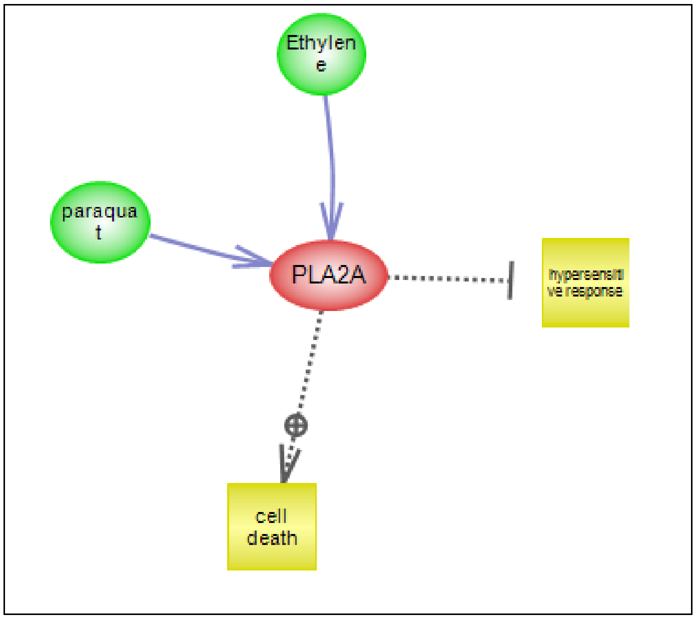

Figure6. Network generation using pathway studio software version 7.1, network shows the interaction between transcripts involved in storage during plant defense, identified in chickpea after 48h post infection with Foc1.

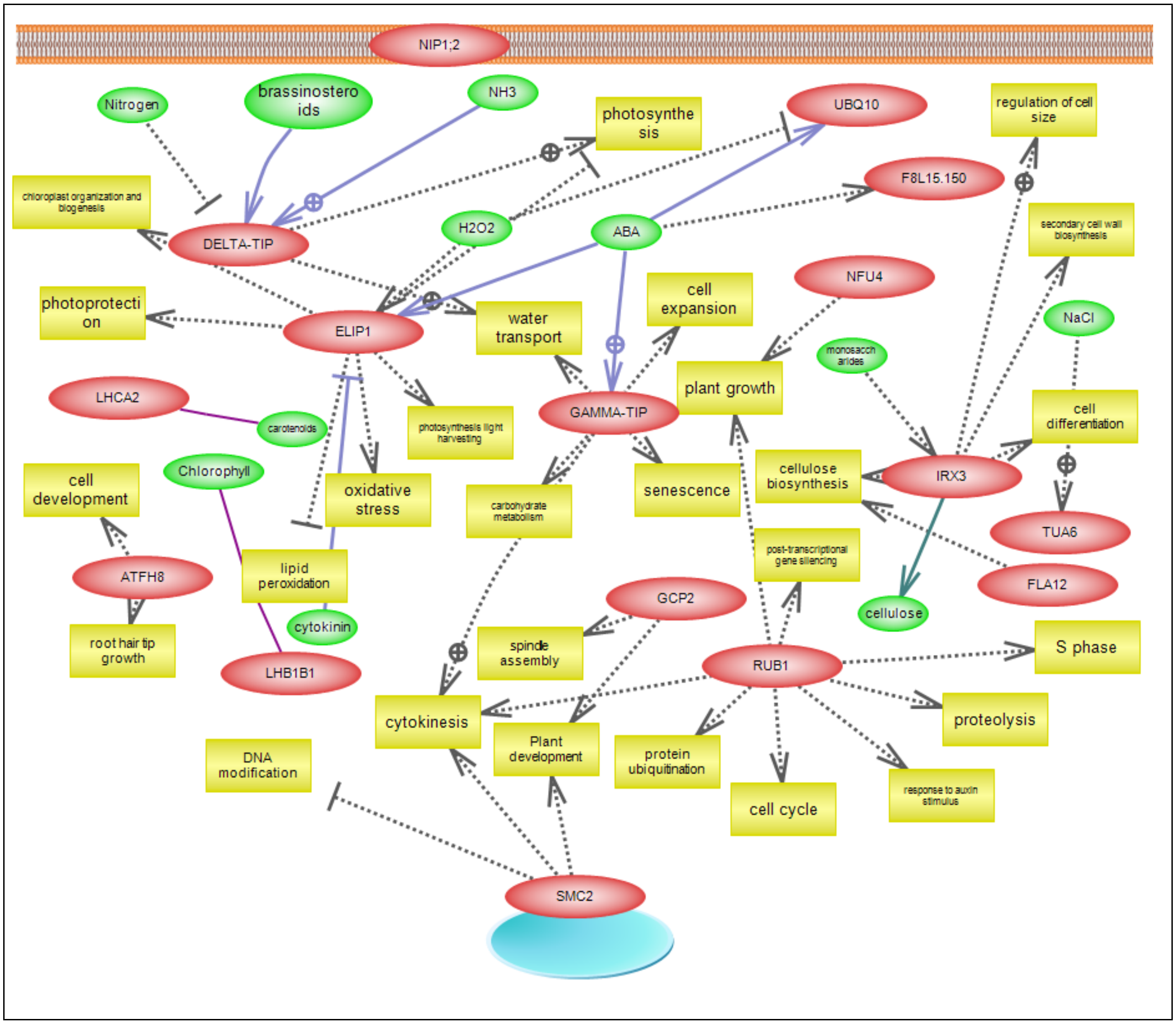

Figure7. Network generation using pathway studio software version 7.1, network shows the interaction between structural transcripts involved in plant defense, identified in chickpea after 48h post infection with Foc1.

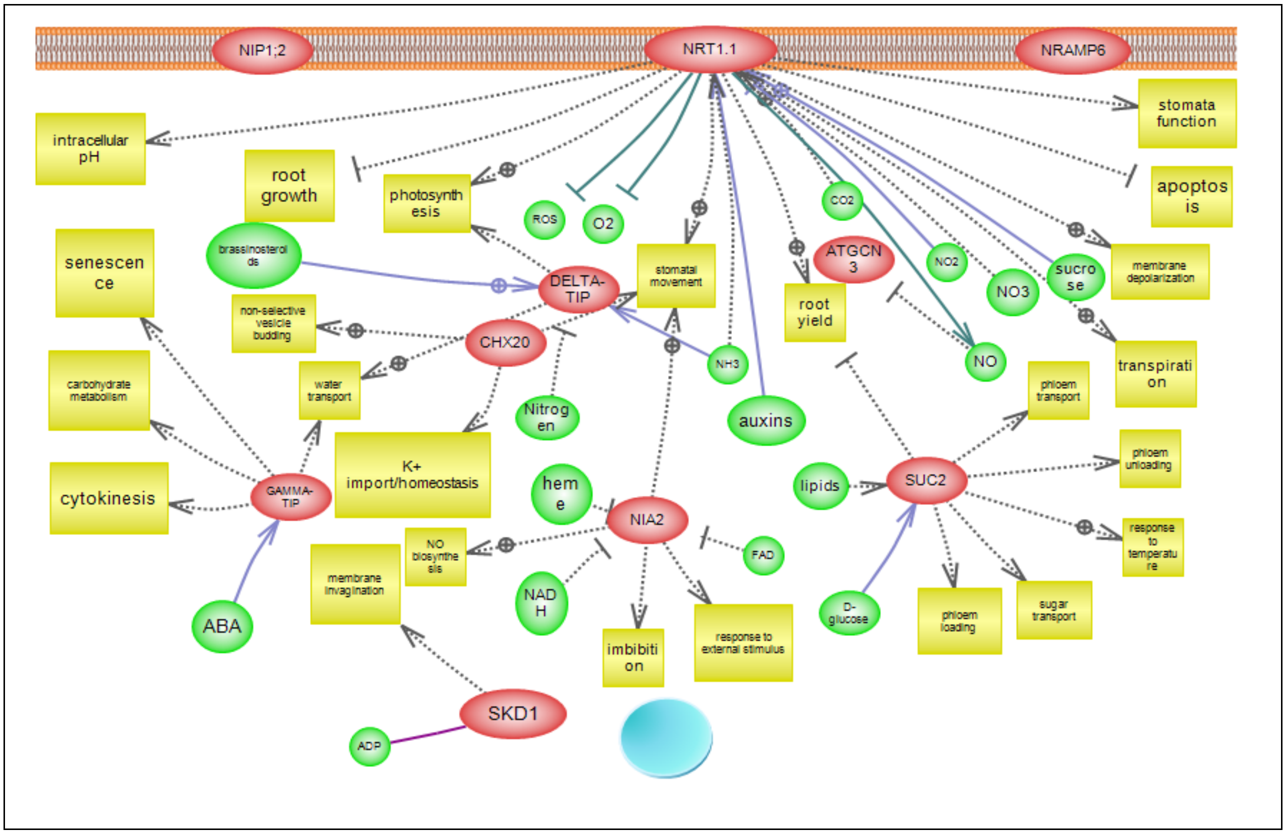

Figure8. Network generation using pathway studio software version 7.1, network shows the interaction between transport-related transcripts involved in plant defense, identified in chickpea after 48h post infection with Foc1.
